# *Zika virus* Pathogenesis and Targeted Intervention Strategies

**DOI:** 10.3390/v18101085

**Published:** 2026-10-01

**Authors:** Liangpeng Suo, Haizhen Fan, Qiong Wu, Xingyang Wei, Hanxi Xiao, Beibei Fu, Xuhu Mao, Xiaoyuan Lin

**Affiliations:** 1 Department of Clinical Microbiology and Immunology, College of Pharmacy and Medical Laboratory, Army Medical University (Third Military Medical University), Chongqing 400038, China; suoliangpeng@tmmu.edu.cn (L.S.); 15774993408@163.com (Q.W.); w_xy_serendipity@163.com (X.W.); m15320716036@163.com (H.X.); fubeibei@tmmu.edu.cn (B.F.); maoxh2012@hotmail.com (X.M.); 2 School of Medicine, Chongqing University, Chongqing 400030, China; fanhaizhen2022@163.com

**Keywords:** *Zika virus* (ZIKV), viral proteins, pathogenesis, type I interferon (IFN-I) pathway, antibody-dependent enhancement (ADE), vaccines, antiviral drugs

## Abstract

*Zika virus* (ZIKV), a mosquito-borne member of the *Flaviviridae* family, is primarily transmitted via the bite of *Aedes aegypti* mosquitoes but can also be spread through sexual contact and vertical transmission. Infection with ZIKV causes Zika fever and Guillain–Barré syndrome (GBS); more importantly, maternal infection during pregnancy can result in severe neurodevelopmental disorders, such as fetal microcephaly. Although the World Health Organization declared the Zika outbreak a Public Health Emergency of International Concern (PHEIC) in 2016, and extensive research has since been conducted, no approved vaccines or therapeutic drugs are currently available. Recent studies have shown that various ZIKV-encoded proteins participate in host-barrier breaching, regulation of tissue tropism, interference with neurodevelopment, mediation of neurotoxicity, and evasion of type I interferon (IFN-I) immune responses, thereby advancing the development of vaccines and antiviral drugs targeting viral proteins. However, the complete mechanisms by which viral proteins synergistically mediate pathogenesis, the clinical significance of antibody-dependent enhancement (ADE) and its prevention and control strategies, and the practical barriers to clinical translation of protein-targeted interventions have not been systematically elucidated. This review is structured around viral proteins as a central framework: it summarizes the molecular mechanisms by which multiple ZIKV proteins synergistically regulate pathogenic processes; reviews the current research basis and response strategies for flavivirus ADE, and outlines unresolved scientific questions; and discusses progress in vaccines and antiviral drugs targeting viral proteins, with emphasis on evidence gaps and clinical translation barriers. By integrating existing findings and unresolved scientific questions, this review provides a research-gap-oriented theoretical reference for basic ZIKV research and clinical translation of prevention and control strategies.

## 1. Introduction

*Zika virus* (ZIKV) is a mosquito-borne flavivirus with diverse and complex transmission routes, including bites by *Aedes aegypti* mosquitoes, sexual contact, and vertical transmission [1]. ZIKV infection not only causes Zika fever and Guillain–Barré syndrome (GBS) in adults but also severely affects fetal neurodevelopment, leading to microcephaly and a spectrum of neurodevelopmental disorders [2,3]. Since its initial isolation from a rhesus monkey in Uganda in 1947, ZIKV maintained low-level endemic circulation for decades [4]. The large-scale epidemic that erupted in Brazil in 2015 and swept across South and Central America was driven by multiple converging factors: the universal lack of pre-existing immunity in human and nonhuman primate populations, abundant *Aedes aegypti* vector populations, and an infection base sufficient to sustain urban transmission. These factors, compounded by increased global travel and trade, facilitated the rapid geographic expansion of ZIKV [5,6]. In 2021, the virus underwent a small-scale resurgence in Kanpur, Uttar Pradesh, India, and it remains endemic in 87 tropical and subtropical countries worldwide [7]. The World Health Organization declared the ZIKV outbreak a Public Health Emergency of International Concern during 2015–2016; the virus now exhibits low-level endemic circulation, and sustained surveillance together with proactive preparedness for prevention and control remains imperative [8]. In recent years, extensive basic research on ZIKV has focused on the structural proteins—envelope protein (E), capsid protein (C), and pre-membrane protein (prM)—and the non-structural proteins: non-structural protein 1 (NS1), non-structural protein 2A (NS2A), non-structural protein 2B (NS2B), non-structural protein 3 (NS3), non-structural protein 4A (NS4A), non-structural protein 4B (NS4B), and non-structural protein 5 (NS5) encoded by its genome [9,10]. Key amino acid mutations in the E [11], prM [12,13], NS2A [14,15], and NS5 [16] proteins of ZIKV, as well as variation in N-linked glycosylation at Asn154 of the E protein, are among the contributing factors to the virulence discrepancy between early African endemic strains and extra-African outbreak strains [17]. These proteins have been established as both key pathogenic effectors and primary targets for vaccine and antiviral drug development [18,19,20]. However, despite decades of intensive research, no licensed ZIKV vaccine or specific antiviral agent is currently available for clinical use globally [19,21].

Nevertheless, substantial knowledge gaps remain in the mechanistic understanding and translational application of ZIKV [18,19,20]. The pathogenic regulatory networks formed by the coordinated action of multiple structural and non-structural viral proteins in vivo have not been fully elucidated [22]. Most existing studies have analyzed individual ZIKV proteins independently, whereas the dynamic cooperation between structural and non-structural proteins in disrupting host barriers, modulating viral tissue tropism, and antagonizing host immune responses during authentic infection remains to be systematically elucidated [18,19,20]. Of particular importance, the molecular basis by which ZIKV preferentially targets neural progenitor cells (NPCs) and disrupts brain development remains unclear [23]. In translational application, antibody-dependent enhancement (ADE) is the major bottleneck in ZIKV vaccine development, and the immune interplay between ZIKV and dengue virus (DENV) has not been fully elucidated [24]. In vitro studies have demonstrated that cross-reactive antibodies at sub-neutralizing levels produced by prior DENV infection can mediate ADE of ZIKV infection via Fcγ receptor pathways [24]. However, multiple prospective cohort studies have shown that pre-existing DENV immunity does not uniformly exacerbate ZIKV disease: high-titer anti-DENV antibodies can confer cross-protection against symptomatic ZIKV infection [25,26]. Currently, how antibody titers, antibody affinity, and other factors jointly determine the outcome (cross-protection vs. ADE) remains unclear [27]. These issues substantially increase the difficulty of rational ZIKV vaccine design and pose safety concerns for broad-spectrum flavivirus vaccines [27]. In addition, although numerous candidate drugs targeting viral structural and non-structural proteins have shown favorable activity in immortalized cell lines and immunodeficient rodent models, anti-ZIKV drug development still faces prominent translational challenges [28]. Most candidate molecules have yet to undergo systematic evaluation of pharmacokinetics, blood–brain barrier penetration, or reproductive toxicity [29]. The highest disease burden of ZIKV occurs during pregnancy, yet early drug development pipelines rarely incorporate teratogenicity and placental transfer assessments [29,30]. Moreover, single-target antiviral strategies carry the risk of viral escape mutations, and the clinical potential of combination regimens remains unvalidated [28]. Nonhuman primate large-animal platforms are urgently needed to bridge the gap between cell/rodent studies and human clinical trials [29].

Therefore, this review focuses on ZIKV-encoded proteins as the central framework and systematically integrates current evidence. First, we describe the synergistic molecular mechanisms by which structural and non-structural proteins mediate key pathogenic events. We then dissect the molecular basis of ADE and systematically summarize vaccine development strategies aimed at mitigating ADE risk. Furthermore, we critically evaluate preclinical anti-ZIKV drug candidates targeting structural and non-structural proteins and analyze the translational barriers confronting antiviral therapies. Ultimately, this review aims to provide a systematic, viral protein-centered theoretical reference for in-depth investigation of ZIKV pathogenesis and the clinical translation of prevention and control strategies.

## 2. ZIKV Proteins and Their Roles in Pathogenesis

**Figure 1:**
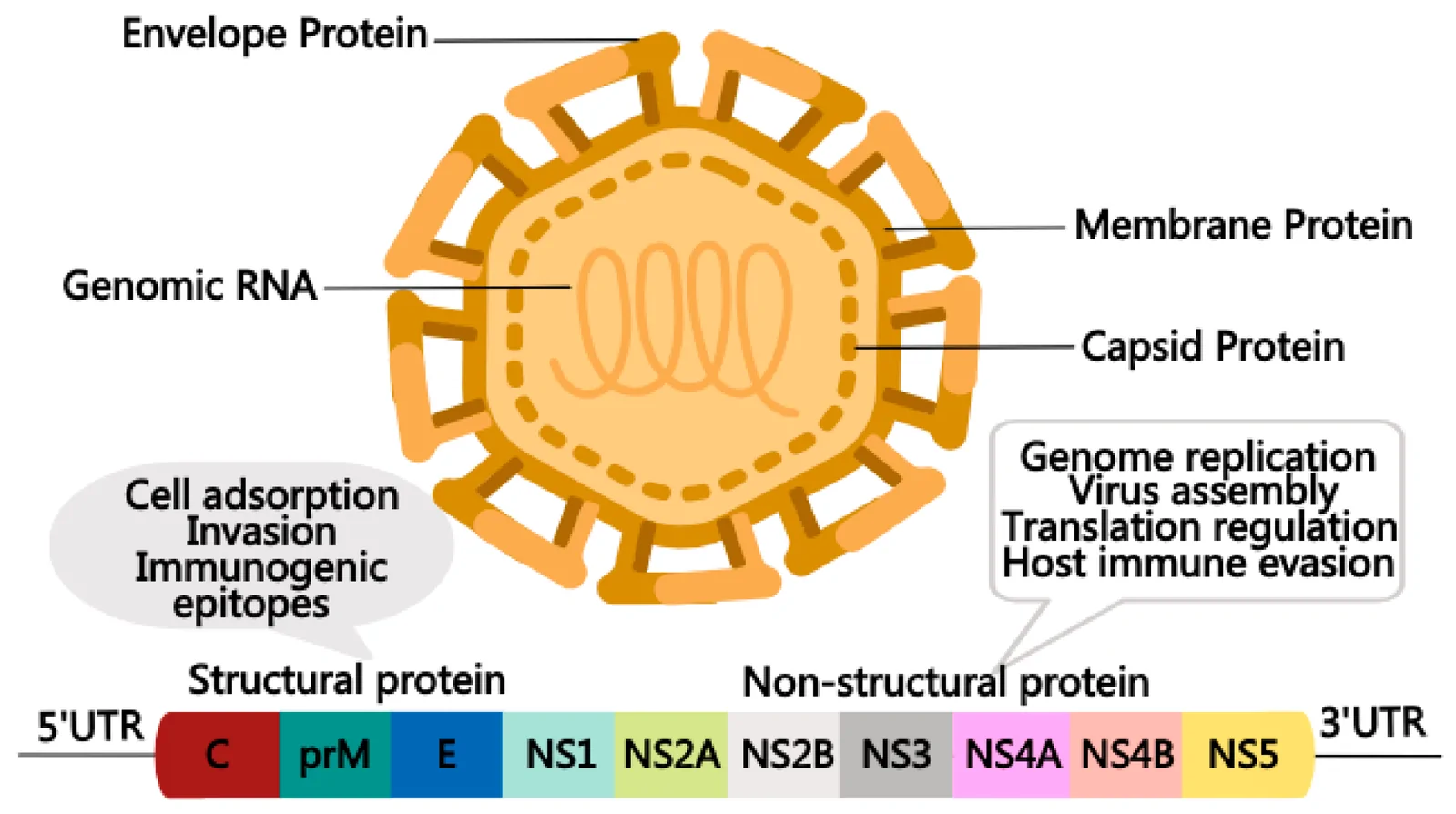
**ZIKV structure and protein functions.** The black lines represent the 5′ and 3′ untranslated regions (UTRs). ZIKV contains a single-stranded positive-sense RNA, which encodes 3 structural proteins (C, prM/M, and E) and 7 non-structural proteins (NS1–NS5). Structural proteins play major roles in cell attachment, viral entry, and providing immunogenic epitopes, while non-structural proteins function in viral replication, assembly, translational regulation, and host immune evasion. Created with MedPeer (medpeer.cn).

ZIKV structural proteins mediate viral attachment, cellular entry, and membrane fusion, and contain the major immunogenic epitopes, while non-structural proteins orchestrate viral genome replication, virion assembly, translational regulation, and modulation of host immune responses (Figure 1). Accumulating direct evidence over the past decade has established that individual ZIKV proteins play critical, non-redundant roles in the pathogenesis of ZIKV-associated diseases via specific physical and functional interactions with a wide array of host cellular factors [3].

### 2.1. ZIKV-Related Neurological Complications

Congenital Zika syndrome (CZS) is associated with a spectrum of brain abnormalities caused by ZIKV crossing the placental barrier and the fetal blood–brain barrier (BBB) to target neural progenitor cells. These abnormalities include microcephaly, parenchymal calcifications, malformations of cortical development, neuronal migration defects, corpus callosum anomalies, ventriculomegaly, and brainstem and cerebellar abnormalities [31]. ZIKV is also strongly linked to GBS in adults, an acute immune-mediated peripheral neuropathy characterized by the core clinical features of flaccid quadriparesis and areflexia [32]. During the 2015–2016 ZIKV epidemic in Brazil, clinical and epidemiological studies first established a definitive etiological association between ZIKV infection and GBS [32]. Beyond these conditions, ZIKV may also cause other neurological complications, including acute myelitis and meningoencephalitis [33].

### 2.2. Breaching of Host Barriers

The ability of ZIKV to traverse host barriers, primarily the placental barrier and the blood–brain barrier, is the core determinant of the severe clinical outcomes it induces [22].

As a key molecule initiating viral infection, the E protein has been confirmed to mediate viral attachment and membrane fusion with host cells [34]. Previous studies using virus binding assays and peptide competition assays have verified that amino acids 268–273 in the DII domain of the E protein are critical for viral attachment [35]. Additionally, the V473M mutation in the E protein of epidemic strains has been shown in animal models to significantly enhance both the intrauterine transmission efficiency of the virus and maternal viremia levels, both of which are thought to directly increase the risk of fetal infection [11]. Most importantly, the E protein can directly disrupt the structural integrity of the blood–brain barrier, increase its permeability, and simultaneously upregulate the expression of leukocyte chemokines and pro-inflammatory factors, thereby activating the inflammatory response in astrocytes [36]. After the E protein initiates infection, NS1 is thought to further amplify the virus’s trans-barrier invasive potential through its barrier-damaging effects. Existing studies have shown that NS1 can induce tissue-specific barrier dysfunction through its wing domain and β-ladder domain, creating a permissive environment for viral invasion [37]. Meanwhile, NS1 can significantly disrupt endothelial barrier integrity by regulating microRNAs (miRNAs), for example, by modulating the expression of cadherin and claudin-5 [38,39] and impairing the stability of β-catenin and vascular endothelial (VE)-cadherin, leading to endothelial barrier dysfunction and increased vascular permeability [40]. NS1 can also induce the formation of tunneling nanotubes (TNTs) and be transferred to adjacent uninfected cells via these structures [41].

### 2.3. Modulation of Viral Tissue Tropism

ZIKV exhibits distinct cellular tropism in vivo, preferentially infecting neural progenitor cells, germ cells, and placental trophoblast cells [42]. This characteristic is a critical prerequisite for the virus to cross host barriers, invade neural tissues and trigger developmental impairments, and establish persistent infection in the host.

Regarding the mechanism of host cell invasion, flaviviruses rely primarily on their envelope E protein to mediate host cell recognition and entry. The E protein binds directly or indirectly to C-type lectin molecules (e.g., DC-SIGN, L-SIGN) and diverse phosphatidylserine (PS) receptors on the host cell surface, thereby mediating viral adsorption, membrane fusion, and endocytosis [10,43]. Such receptor redundancy markedly expands the range of cells susceptible to ZIKV infection and also provides a molecular basis for its tropism toward the aforementioned key target cells.

Furthermore, post-translational modifications of the E protein critically regulate ZIKV tissue tropism. The E protein acts as a substrate for the host E3 ubiquitin ligase tripartite motif-containing protein 7 (TRIM7) and undergoes K63-linked polyubiquitination. This modification is a critical regulatory step in determining ZIKV tissue tropism [44]. Under the tested experimental conditions, ZIKV mutants lacking N-glycosylation at the Asn154 residue of the E protein display significantly reduced viral loads in serum, eyes, and brain tissues of infected mice, suggesting that E protein glycosylation at this site is indispensable for efficient viral replication and dissemination in neural tissues and immune-privileged sites [45].

Notably, ZIKV exhibits a distinct receptor utilization preference within the flavivirus genus, enabling it to exploit the AXL receptor for highly efficient cellular entry. Specifically, ZIKV binds to growth arrest-specific gene 6 (GAS6), a specific ligand of AXL, via exposed phosphatidylserine (PS) on the viral envelope; GAS6 then mediates the interaction between the virus and cell-surface AXL, initiating downstream endocytic signaling to drive cell entry [46]. In sharp contrast, DENV and West Nile virus (WNV) do not utilize the AXL receptor as a primary entry factor, representing a major molecular driver underlying the substantial disparities in tissue tropism and pathogenic phenotypes between ZIKV and these two flaviviruses [47,48]. Correspondingly, the AXL receptor is highly expressed in neural stem cells, germ cells, and placental trophoblast cells [42,49], and this expression pattern aligns closely with ZIKV tissue tropism, further explaining why ZIKV can specifically target these cells to establish efficient infection and cause tissue damage. Contrary to the assumption that AXL is an indispensable entry receptor, accumulating evidence from multiple model systems has demonstrated its dispensability for ZIKV infection. Intracranial inoculation of AXL-knockout mice did not reduce viral replication in brain tissue, indicating that AXL is not essential for neural infection in the brain [50]. Similarly, simultaneous ablation of AXL, MERTK, and TYRO3—all members of the TAM receptor family—failed to completely block ZIKV infection following subcutaneous, intravaginal, intraperitoneal, or intracranial challenge in mice [51]. In human neural progenitor cells and brain organoids, CRISPR-mediated AXL knockout also failed to confer resistance to ZIKV [23]. Collectively, these findings support the existence of a redundant receptor network for ZIKV entry, comprising C-type lectins (DC-SIGN and L-SIGN), phosphatidylserine receptors (TIM-1 and TIM-4), and additional, as-yet-unidentified receptors.

Recent studies have further revealed that ZIKV actively modulates cell-surface receptor abundance through host trafficking pathways. In epithelial cells, glioblastoma cells, and stem cell-derived trophoblasts, ZIKV infection induces autophagy via the E, NS2A, NS3, and NS4B proteins, leading to downregulation of AXL and TIM-1 [52]. Mechanistically, viral proteins promote lysosomal degradation of entry receptors, thereby preventing superinfection, limiting cell death, and sustaining long-term viral replication. In trophoblasts, AXL downregulation attenuates excessive placental inflammation without preventing fetal brain infection, suggesting that dynamic receptor modulation serves as an immune evasion strategy rather than merely determining cellular susceptibility [52].

In addition, host innate immune genetic variation, particularly in fetuses and neonates, has been associated with congenital CZS severity. CZS patients carrying the *CXCL8* rs4073 T allele or the *TLR7* rs179008 T allele, both functionally annotated as low-expression/low-product variants, exhibit a significantly increased risk of severe microcephaly (OR ≈ 2.36 and 3.66, respectively). Maternal *TREM1* (rs2234246 C) and IL4 (rs2243250 C) variants, together with offspring *CXCL10* (rs4508917 G) and *CXCL8* variants, have been linked to CZS risk [53]. Other studies have reported that the neonatal IFNAR1 rs2257167 CG/CC genotype increases CZS risk by approximately 3.4-fold [54,55].

Accordingly, CZS severity appears to be governed by the combined effects of receptor redundancy, cell-type-specific receptor expression, virus-mediated dynamic receptor modification, and host innate immune genetic variation. Nevertheless, current evidence remains limited. Receptor-related regulatory mechanisms have been mainly established in cell, animal, and organoid models, whereas association analyses of host genetic variants are largely restricted to small, single-population cohorts. Large-scale, multi-population clinical validation and causal functional experiments are still lacking, and further investigations are required to substantiate these findings.

### 2.4. ZIKV Proteins Interfere with Neural Development

Multiple ZIKV proteins directly target key steps in neurogenesis, thereby fundamentally disrupting brain development. The C protein is regarded as one of the core regulators that perturb neural stem cell differentiation programs. It specifically binds to and inhibits Dicer, blocking miRNA biogenesis in neural stem cells, while also disrupting the nonsense-mediated mRNA decay (NMD) pathway; these two effects together trigger neurodevelopmental defects [56,57]. It also downregulates Numb, a key neurogenesis protein, in a dose-dependent manner, impairing asymmetric cell division of neural stem cells [58]. Furthermore, it globally downregulates neurodifferentiation-associated pathways, including axon guidance, Wnt, MAPK, and TGF-β signaling, while upregulating the p53 apoptotic pathway, thereby impairing neurodifferentiation at multiple levels [59]. In addition, the high thermal stability and N-terminal autoinhibitory conformation of the C protein regulate its lipid-binding activity, which may indirectly disrupt lipid metabolic homeostasis in neural stem cells and modulate their differentiation [60].

The NS2B-NS3 complex specifically cleaves Septin-2 in human NPCs, directly inhibiting cytokinesis, triggering genotoxic stress and cell death, and depleting the cellular pool for neurogenesis [61]. NS4A and NS4B have been reported to synergistically disrupt neurogenesis: NS4A directly binds to ANKLE2, a key microcephaly-associated protein, and blocks its downstream signaling, a potential mechanism underlying the microcephaly phenotype [62,63]. These two proteins jointly dysregulate the Akt-mTOR pathway, inhibiting neurogenesis and inducing autophagy [64]. When co-expressed in fetal neural stem cells, they further exacerbate autophagy and impair the Notch pathway, arresting cell differentiation [65]. The interaction between NS4B and VCP also aggravates autophagy dysregulation, thereby synergistically promoting neurogenesis abnormalities [66]. As a global transcriptional regulator, NS5 directly binds to neurodevelopmental gene loci and blocks RNA polymerase II-associated factor 1 complex (PAF1C)-mediated transcriptional elongation, globally repressing the transcription of neurodevelopment-related genes [67]. In addition, NS5 regulates mitosis via the destruction box (D-box) and disrupts microtubule homeostasis, blocking core neurogenesis programs at both the transcriptional and cell division levels [68,69].

### 2.5. ZIKV Protein-Mediated Neurotoxicity

In addition to disrupting neurogenesis programs, multiple viral proteins can amplify neural cell damage through distinct pathways, driving irreversible lesion progression. The E protein induces overexpression of poly(ADP-ribose) polymerase 1 (PARP1) in neurons, activating PARP1-dependent cell death and causing neuronal injury [70]. The deglycosylated viral strain exhibits increased replication capacity in neural tissue, thereby further upregulating PARP1 and exacerbating apoptosis [71]. NS1 inhibits the antioxidant response by upregulating Bach1, leading to reactive oxygen species (ROS) accumulation and aggravating oxidative damage [72].

Meanwhile, NS1 induces the formation of neutrophil extracellular traps (NETs), causing secondary neural tissue damage [73]. Its glycosylation and ubiquitination modifications regulate replication compartment formation and host adaptation, respectively, collectively amplifying intracranial damage [74,75].

NS2A binds to sarco/endoplasmic reticulum Ca^2+^-ATPase (SERCA) to disrupt calcium homeostasis, inducing endoplasmic reticulum stress and exacerbating NPC apoptosis [76]. NS2A also degrades neuroepithelial junction proteins, disrupting the laminar structure of the cerebral cortex. Furthermore, it interferes with testosterone synthesis by inhibiting CYP17A1 translation, further aggravating brain developmental abnormalities through endocrine dysregulation [77]. NS4B recruits Bax to mitochondria to trigger apoptosis, resulting in the loss of NPCs and neurons [78].

### 2.6. ZIKV Proteins Mediate Inhibition of the Type I Interferon (IFN-I) Pathway

**Figure 2:**
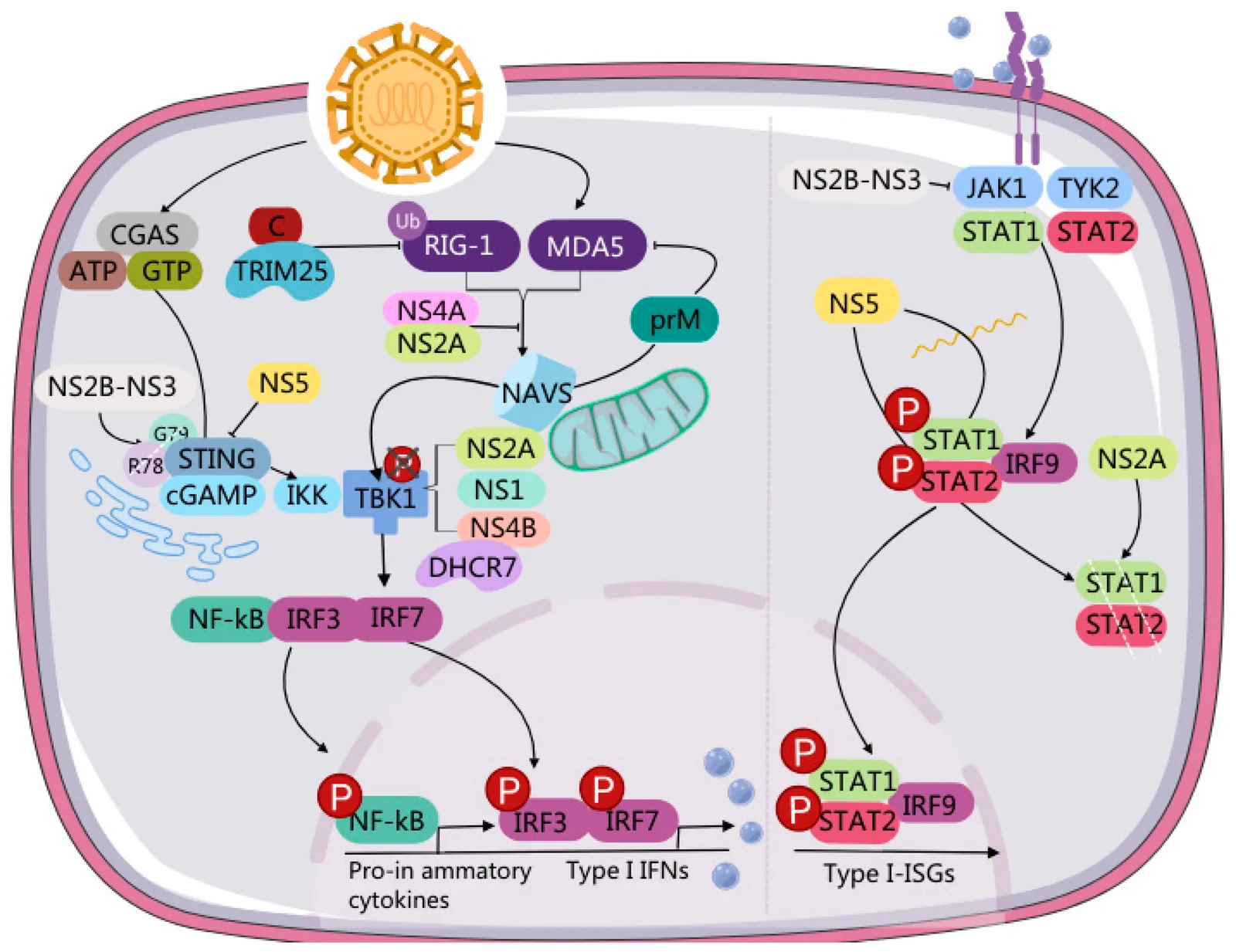
**Molecular mechanism of ZIKV blocking the IFN-I pathway at multiple nodes via synergistic action of multiple proteins.** ZIKV-encoded structural and non-structural proteins exert synergistic blocking effects throughout the IFN-I pathway. In the upstream IFN-I production phase, C protein exerts source inhibition by suppressing TRIM25-mediated RIG-I ubiquitination and downregulating PEX19 to reduce peroxisomes; prM protein binds MDA5 and MAVS to block the RLR pathway; non-structural proteins NS2A, NS2B/3, NS4A, NS1, and NS4B target key molecules, including MDA5/RIG-I, STING, MAVS, TBK1, and IRF3, which form multiple blockades at the corresponding RLR-MAVS, cGAS-STING, and TBK1-IRF3 signaling axes; the methyltransferase domain of NS5 binds STING to inhibit the terminal transcription of IFN-β. In the downstream JAK-STAT effector phase, NS5 and NS2A synergistically target the phosphorylation and proteasomal degradation of STAT1/STAT2, systematically blocking the expression of ISGs and ultimately achieving immune evasion. Created with MedPeer (medpeer.cn).

ZIKV-encoded structural and non-structural proteins exert synergistic inhibitory effects on the host IFN-I production pathway at multiple hierarchical levels, including innate immune sensing, signalosome assembly, kinase signaling cascades, and transcriptional activation, which ultimately suppresses IFN-I production and lays the foundation for viral replication, subsequent host barrier penetration, and neural damage induction [79,80] (Figure 2).

#### 2.6.1. ZIKV Proteins Inhibit the IFN-I Production Pathway

At the most upstream stage of innate immune sensing and pathway initiation, structural proteins mediate the earliest inhibitory effects. The C protein directly binds to tripartite motif-containing protein 25 (TRIM25) to suppress RIG-I ubiquitination and activation, and simultaneously downregulates peroxisome levels by binding to peroxisomal biogenesis factor 19 (PEX19), thereby blocking the initiation of IFN transcription at the viral RNA recognition stage [81,82].

Following the blockade of upstream immune sensing pathways, ZIKV structural and non-structural proteins further target the pivotal RLR-MAVS signaling axis. Specifically, the prM protein interacts with core RLR pathway components MDA5 and MAVS to efficiently suppress the complete activation of the RLR pathway [83]; NS2A blocks the interferon response by inhibiting key factors in the MDA5/RIG-I signaling pathway, thereby reducing nuclear factor kappa-B (NF-κB) promoter activity and suppressing TANK-binding kinase 1 (TBK1) phosphorylation [84,85]. For the cyclic GMP-AMP synthase (cGAS)-stimulator of interferon genes (STING) pathway, the NS2B/3 protease complex cleaves human STING (hSTING) at arginine 78 (R78) and glycine 79 (G79) within its cytoplasmic loop, thereby directly inhibiting the cGAS/STING pathway and impairing host cell intrinsic antiviral defense [86]. NS4A synergizes with NS2A to markedly inhibit MDA5/RIG-I-mediated NF-κB activation [87]. NS4A potently inhibits MAVS-dependent IFN-I responses by disrupting the interaction between RLRs and MAVS and by inducing mitochondrial fission and mitophagy [88]. At the TBK1–interferon regulatory factor 3 (IRF3) phosphorylation cascade, NS1 blocks downstream signal transduction by inhibiting TBK1 phosphorylation and interfering with RLR signalosome assembly [65,89]. NS4B binds to 7-dehydrocholesterol reductase (DHCR7), a key enzyme in cholesterol metabolism, to suppress phosphorylation of both TBK1 and IRF3. Furthermore, NS1 and NS4B exert a synergistic inhibitory effect on IFN-β signal transduction at the TBK1 level, providing complementary mechanisms for pathway blockade [65,90].

At the terminal stage of IFN-β transcriptional activation, the methyltransferase (MTase) domain of NS5 directly interacts with STING to restrict STING-induced IFN-β expression, with the conserved active-site residue D146 playing a critical role in this process [91]. The synergistic action of these multiple proteins at distinct pathway nodes enables comprehensive inhibition of host IFN-I production from the initial sensing step to the terminal transcriptional process.

#### 2.6.2. ZIKV Proteins Antagonize IFN-I Downstream Signaling Pathway

Following IFN-I secretion, ZIKV also blocks JAK-STAT signal transduction at multiple key nodes through the synergistic action of several proteins, thereby preventing the establishment of the host antiviral state. Among these, NS5 is the primary effector that antagonizes downstream IFN-I signaling. On the one hand, NS5 cooperates with ZIKV non-coding RNAs to inhibit the phosphorylation of signal transducer and activator of transcription 1 (STAT1), directly blocking STAT activation and signal transduction [92]. On the other hand, NS5 targets signal transducer and activator of transcription 2 (STAT2) for proteasome-dependent degradation, effectively paralyzing the pathway at the level of key signaling molecule stability [93,94]. NS2A directly mediates the proteasomal degradation of both STAT1 and STAT2, thereby providing functional redundancy [95]. In addition, the NS2B/3 protease suppresses the activation of the JAK-STAT pathway to attenuate RLR-mediated apoptosis, thereby prolonging the survival of infected cells and supporting the completion of the viral life cycle and persistent viral proliferation [96].

## 3. Vaccines: Current Challenges and Potential Strategies

ZIKV vaccine development encompasses both traditional platforms, such as inactivated and live-attenuated vaccines, and novel mainstream platforms, including mRNA, DNA, viral vector, virus-like particle (VLP), and recombinant subunit vaccines. These platforms exhibit substantial differences in development progress, immunogenicity, safety, and technical characteristics (Appendix A).

Despite extensive research efforts, no ZIKV vaccine has been approved for clinical use globally to date. In addition to efficacy and other factors, the risk of ADE remains a major bottleneck impeding ZIKV vaccine development. ADE, first documented in DENV infection in 1977, has long been a core scientific challenge in flavivirus prevention and control [97]. ZIKV and DENV are closely related flaviviruses that exhibit extensive genomic and protein structural homology, co-circulate in endemic regions, and can infect the same individuals [98,99]. Conserved fusion loop epitopes (FLEs) within the E protein of both viruses elicit cross-reactive antibodies with limited neutralizing activity, representing a principal driver of ADE [100]. In individuals with pre-existing DENV immunity who are subsequently infected with ZIKV, or following immunization with immunogens harboring intact conserved epitopes, subneutralizing or non-neutralizing antibodies may be generated. These antibodies bind virions through their antigen-binding sites to form stable virus–antibody immune complexes (V-Ab ICs). Via Fc-mediated engagement of Fcγ receptors (FcγRs), V-Ab ICs markedly enhance viral uptake by target cells compared with antibody-free conditions [101]. Monocytes, macrophages, and dendritic cells—the principal target cells of flaviviruses—express FcγRs [102]. Following FcγR engagement, downstream signaling initiates a self-perpetuating cycle of immune suppression and inflammatory injury. On one hand, signaling through RIG-I, MDA5, and STING pattern recognition receptors (PRRs) is inhibited, thereby reducing type I interferon (IFN-I) production and attenuating the host antiviral state [103]. On the other hand, secretion of the anti-inflammatory cytokine interleukin-10 (IL-10) is robustly induced, accompanied by upregulated expression of suppressors of cytokine signaling (SOCS1 and SOCS3). Elevated IL-10 skews immune responses toward a Th2 profile while suppressing Th1-mediated antiviral immunity, further compromising host-specific antiviral defense [104].

Currently, in vitro experiments and animal models have elucidated some molecular mechanisms of ADE, but the clinical significance of this effect in humans remains highly complex. Population epidemiological evidence further indicates that the disease-enhancing effect of ADE is not absolute; rather, it is modulated by multiple factors, including prior infection history, temporal dynamics of antibody titers, and viral serotype combinations, reflecting a non-reciprocal cross-immunity characterized by the coexistence of protection and enhancement [26,27,105]. On the one hand, prior ZIKV infection significantly increases the risk of subsequent severe dengue: compared with individuals with pre-existing flavivirus antibodies, those with primary ZIKV infection have a markedly higher risk of subsequent symptomatic DENV-2, DENV-3, and DENV-4 infections and dengue with warning signs/severe dengue (DwWS/SD) [27]. On the other hand, prior DENV infection can confer cross-protection against ZIKV. A long-term pediatric cohort study in Nicaragua by the Harris group demonstrated that cross-reactive antibodies induced by prior DENV infection exert titer-dependent bidirectional effects: high titers provide cross-serotype protection, whereas after waning to intermediate levels over time, heterologous serotype re-exposure significantly increases the risk of severe dengue. This study also revealed asymmetry in immune interactions among serotypes (antibody waning after DENV-1 infection and secondary DENV-2 infection being associated with the most prominent increase in severe disease risk), indicating that infection interval and antibody decay kinetics are key variables determining whether ADE manifests at the population level [25,105]. Similarly, the Ko team confirmed that individuals with higher DENV antibody titers have a lower risk of ZIKV infection and disease, suggesting that high-level DENV immunity may be protective rather than infection-enhancing [26]. In summary, in vitro ADE results cannot be directly equated with population disease risk, which has important implications for flavivirus vaccine development and epidemic risk assessment.

In summary, the epidemiological effects mediated by prior flavivirus immunity exhibit pronounced bidirectionality, with final infection outcomes governed by multiple factors, including individual viral exposure history, antibody titers, viral serotype, and cohort characteristics. The relative contributions of ADE-mediated injury and cross-protection to ZIKV and DENV disease outcomes remain incompletely defined. Therefore, large-scale, multi-population prospective studies employing standardized serological and virological endpoints are required to systematically clarify the true clinical significance of ADE, thereby providing a scientific theoretical basis for ZIKV vaccine development and the optimization of clinical therapeutic strategies.

**Figure 3:**
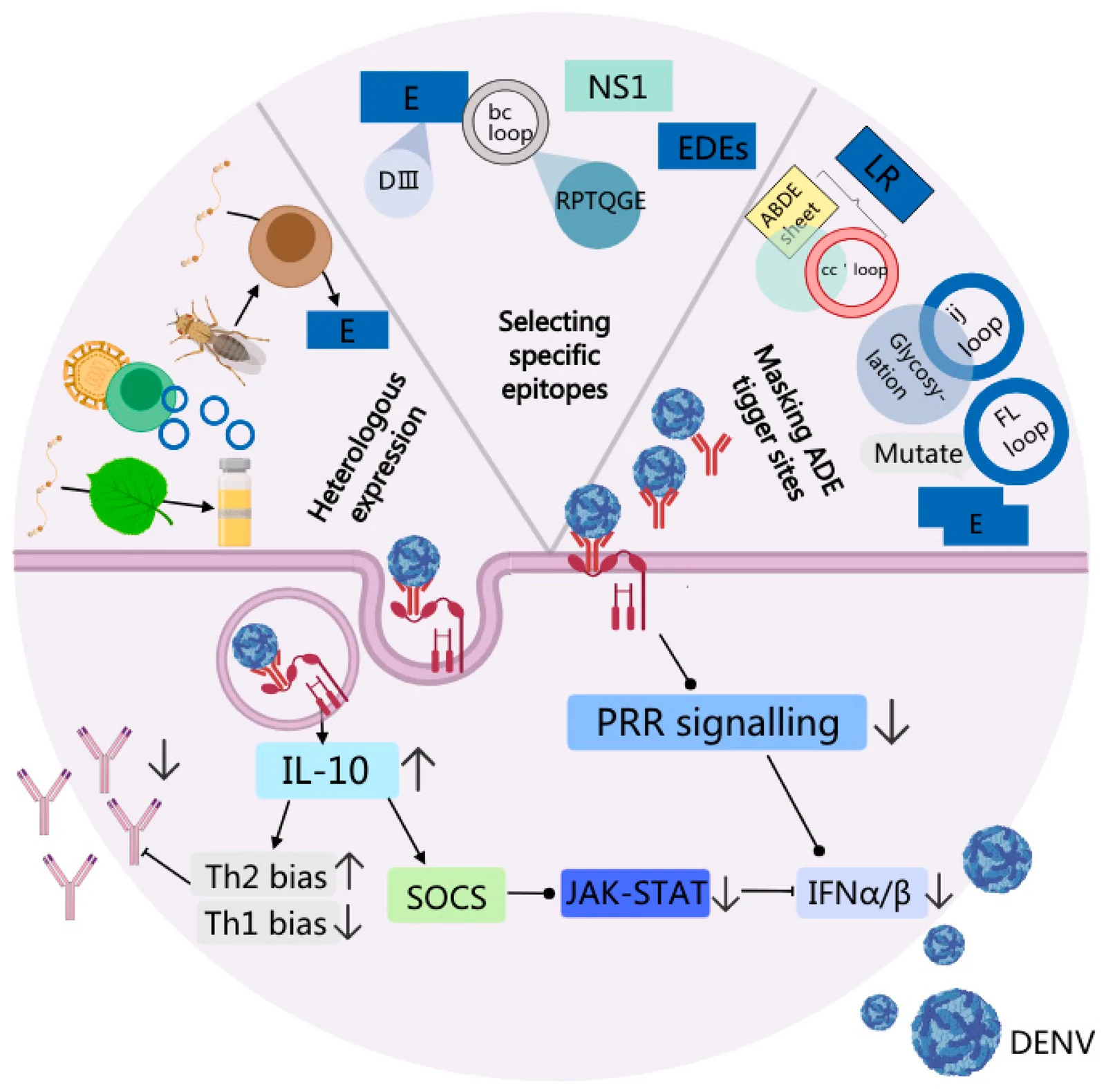
**Mechanisms of ADE formation and mitigation strategies in vaccine development.** Cross-reactive antibodies form immune complexes (V-Ab ICs) with immature non-infectious viral particles. Upon binding to Fc receptors, these complexes inhibit type I interferon release, upregulate IL-10/SOCS, and ultimately trigger the extrinsic ADE pathway. For ADE induced by ZIKV vaccines, mainstream strategies include heterologous antigen expression, specific epitope selection, and modification of ADE-triggering epitopes (left to right Arrows pointing upward (↑) indicate increased expression or enhanced response; arrows pointing downward (↓) indicate inhibition or decreased expression. Abbreviations: E, envelope protein; NS1, non-structural protein 1; PRR, pattern recognition receptor; JAK-STAT, Janus kinase–signal transducer and activator of transcription; IL-10, interleukin-10; SOCS, suppressor of cytokine signaling; IFNα/β, type I interferon; DENV, *dengue virus*.). Created with MedPeer (medpeer.cn).

To address the issue of vaccine-induced ADE, mainstream approaches include modifying key ADE-triggering epitopes, selecting specific epitopes, and utilizing heterologous expression (Figure 3). Among the selected specific epitopes, the most widely used are the EDIII region of the E protein and NS1. These two epitopes exhibit low similarity to those of other flaviviruses and induce highly specific antibodies [106,107,108,109,110,111]. To address the relatively low immunogenicity of these isolated epitopes, carriers or adjuvants can be incorporated to enhance immune responses. For instance, the conjugation of hemoglobin to mannan significantly enhances the immunogenicity of EDIII [112]. Additionally, nanoparticles can be used to enable multimeric antigen display, thereby enhancing both immunogenicity and antigen stability [113,114]. Furthermore, the novel bc loop epitope RCPTQGE (amino acids 73–79) conserved in the E proteins of DENV and ZIKV can induce cross-neutralizing antibodies without triggering ADE in passive immunity models [115]. Mimicking ZIKV and DENV envelope dimeric epitopes (EDEs) induces broad-spectrum neutralizing antibodies without the need for full-length E protein, thereby avoiding the generation of cross-reactive non-neutralizing antibodies. However, free mimetic epitopes are structurally disordered, fail to elicit robust immune responses, and are rapidly cleared in vivo; thus, they require display on adeno-associated virus capsids to restore their native conformation and enable recognition by EDE-specific antibodies [116,117]. VLPs also support multi-epitope antigen display, and integrating both ZIKV and DENV antigens on this platform allows for simultaneous protection against both viruses [118].

Another strategy involves modifying key ADE-triggering epitopes. For example, the EDIII immunogen surface can be engineered using protein engineering techniques: the LR epitope, which serves as the binding site for protective antibodies, is preserved, while binding sites for non-neutralizing or variably neutralizing antibodies (including the ABDE sheet and CC’ loop regions) are masked. This approach significantly reduces the ADE effect while maintaining vaccine efficacy [119]. Mutations in the native FL region of EDII can shift vaccine-induced immunodominance toward ZIKV-specific immunity, thereby eliminating ADE responses while preserving neutralizing antibody titers (1:800 versus 1:1000 for the wild-type E protein). These mutations maintain E protein structural stability and preserve neutralizing epitopes [116]. Glycosylation masking mutations in the ij loop of ZIKV E protein (E-248NHT) and DENV2 E protein (E-242NHT) eliminate cross-reactive antibodies that enhance both DENV and ZIKV infection [120]. Overall, masking and modifying E protein epitopes alone are insufficient to fully eliminate ADE-inducing antibodies [116,121].

Heterologous expression of the E protein effectively reduces ADE effects. Exosomes derived from ZIKV-infected cells are non-infectious, display abundant E protein, and attenuate ADE responses [122]. E protein produced in insect cells with four mutations in the FL region and adjacent areas induces antibodies with neutralizing capacity comparable to that of the wild-type antigen, but with significantly reduced serological cross-reactivity and no detectable in vitro ADE activity [123]. Plant-produced vaccines protect mice from lethal West Nile virus infection without enhancing ZIKV or DENV infectivity [124]. Furthermore, polyclonal avian IgY raised against fully inactivated DENV2 effectively treats DENV infection in mice; because the Fc region of IgY cannot bind to mammalian Fc receptors, it does not induce ADE. Similarly, polyclonal ZIKV-specific IgY provides passive immune protection without triggering ADE, representing a promising novel strategy for the prevention and control of DENV and ZIKV infections [125,126].

ADE risk varies significantly across different vaccine platforms, which directly determines the prioritization of platforms for vaccine development. mRNA vaccines, DNA vaccines, and recombinant subunit vaccines enable precise control over antigen epitope presentation, elicit a high proportion of serotype-specific antibodies, and are associated with low ADE risk [127]. Among these, mRNA vaccines enhance antigen expression efficiency via lipid nanoparticle (LNP) delivery systems while avoiding the formation of intact viral particles, thereby further reducing ADE risk [100]. Notably, circRNA vaccines exhibit greater stability and drive more durable antigen expression [128]. VLP vaccines represent another promising direction for future development, provided that the VLPs are prepared as mature particles with complete prM cleavage and accurately present low-risk epitopes [129]. In contrast, live-attenuated vaccines and whole-virus inactivated vaccines inevitably induce cross-reactive antibodies if they express full-length prM/E proteins. Although attenuation or inactivation reduces the risk of productive infection, these platforms carry higher ADE risk than epitope-defined vaccines and require rigorous clinical safety assessment [109]. The licensed dengue virus vaccine Dengvaxia (CYD-TDV) employs the YFV-17D backbone to express chimeric DENV prM/E proteins, a strategy that can also be applied to ZIKV vaccine development [130].

Epidemiological evidence provides critical guidance for the design of ZIKV vaccine trials, shifting the research paradigm from simple efficacy verification to precise immune background-based risk–benefit evaluation. Specifically, baseline serological status should be regarded as a core criterion for participant enrollment and stratification in trial design. For dengue vaccines, a rigorous screen-before-vaccination strategy is required to avoid ADE risks in seronegative populations. For ZIKV vaccine trials, participants should be stratified according to prior dengue infection history and baseline antibody titers [27]. Furthermore, given that antibody decay kinetics serve as a crucial determinant of ADE occurrence, trials should establish a long-term follow-up period of at least 3–5 years with regular monitoring of neutralizing antibody titers and antibody affinity maturation [131]. Severe disease prevention and hospitalization rates should be defined as key clinical endpoints, and serotype-specific efficacy analyses should be performed to identify potential risk windows caused by asymmetrical immune interactions [25,105]. In addition, booster vaccination strategies should be prospectively explored to sustain protective antibody levels. Meanwhile, an independent data and safety monitoring system should be established to dynamically assess the incidence of severe adverse events in vaccine groups, thereby ensuring the safety and efficacy of candidate vaccines under the complex immune background of real-world endemic settings. Although pregnant women have historically been excluded from vaccine development trials, the disease burden of ZIKV infection is most severe during pregnancy. Recent ethical guidance emphasizes three core imperatives: (i) development of vaccines that are safe and reasonably applicable in pregnancy; (ii) systematic collection of pregnancy-specific safety and immunogenicity data for all candidate vaccines to which pregnant women may be exposed; and (iii) equitable inclusion of pregnant women in clinical trials when risk–benefit assessment is favorable [132,133]. Recruitment of pregnant participants nevertheless faces substantial practical obstacles, including sporadic and unpredictable ZIKV outbreaks that complicate prospective trial design; geographically dispersed populations in affected regions; and the need for enhanced obstetric surveillance, reproductive toxicology studies, and long-term follow-up of infant neurodevelopment [134]. Moreover, ethical inclusion of pregnant women in ZIKV vaccine trials requires a dedicated regulatory framework [135]. Collectively, these factors markedly increase trial cost and complexity. Given declining ZIKV incidence and the infeasibility of large-scale phase III efficacy trials in pregnant women, alternative regulatory strategies are being explored [133]. Immunobridging leverages well-established correlates of protection (CoP) from non-pregnant adults or nonhuman primate (NHP) models to infer vaccine protective efficacy rigorously without placebo use or deliberate maternal exposure to the virus [136,137]. This paradigm shift from conventional efficacy trials toward CoP-centered regulatory science supported by NHP bridging data is essential to accelerate the availability of ZIKV vaccines for populations at high risk of congenital infection [138].

## 4. ZIKV Antiviral Drugs: Focus on Protein Targets

### 4.1. Drugs Related to Structural Proteins

Therapeutic strategies targeting structural proteins are predominantly focused on the ZIKV E protein. Preclinical studies have explored three principal avenues: natural product screening, synthetic small-molecule design, and monoclonal antibody development. Each approach offers distinct advantages and limitations [139].

#### 4.1.1. Inhibitors Targeting the E Protein

Accumulating evidence has demonstrated that natural products, including epigallocatechin gallate (EGCG), curcumin, gossypol, and resveratrol, exhibit potent anti-ZIKV activity. In Vero cell-based viral inhibition assays, the half-maximal inhibitory concentration (IC_50_) values of EGCG, curcumin, and gossypol were 12.5 μM, 8.3 μM, and 25 μM, respectively. The primary mechanism of action of these compounds involves direct binding to the E protein, thereby blocking ZIKV entry into host cells [140,141]. However, these natural products have significant druggability limitations: they exhibit a narrow safety window, as exemplified by EGCG, which reduced Vero E6 cell viability by 20% after 72 h of incubation at 200 μM [140]. Compared with natural products, synthetic small-molecule compounds exhibit more favorable druggability, and screening of synthetic compound libraries has yielded high-quality lead compounds. Using AlphaScreen technology, seven small-molecule inhibitors targeting the ZIKV E protein were identified, which markedly suppressed viral replication at non-cytotoxic concentrations, with 90% inhibitory concentration (IC_90_) values ranging from 5.2 to 20.3 μM. [142]. Structural biology studies further defined domain III (DIII) of the ZIKV E protein, in proximity to Asn154 and the fusion loop, as the optimal drug-binding site, providing a theoretical basis for subsequent medicinal chemistry optimization and rational design of highly active inhibitors [143]. To date, however, systematic in vivo pharmacokinetic and safety evaluations of these synthetic leads remain lacking, leaving a considerable gap to clinical translation. Beyond natural products and synthetic small molecules, drug repurposing is an efficient strategy for anti-ZIKV drug development, leveraging approved drugs with well-established safety data to shorten development timelines and reduce developmental risks [144]. Erythromycin estolate (Ery-Est), a clinically approved macrolide antibiotic, has demonstrated broad-spectrum anti-flaviviral activity, with IC_50_ values of 3.22–7.14 μM against multiple ZIKV strains. Mechanistic studies indicate that Ery-Est dose-dependently disrupts the structural integrity of the ZIKV E protein and blocks E protein-mediated virus–host membrane fusion, thereby inhibiting viral infection and propagation [144,145]. Animal studies further corroborate that Ery-Est confers neuroprotection and effectively protects mice against lethal ZIKV challenge, underscoring its considerable translational potential [144].

Collectively, natural product screening, synthetic small-molecule design, and repositioning of approved drugs constitute three core development pathways for E protein-targeting small-molecule anti-ZIKV therapeutics. Among these, systematic computational screening of approved drug libraries to identify novel antiviral candidates represents an efficient means to rapidly expand the anti-ZIKV therapeutic pipeline.

#### 4.1.2. Neutralizing Antibodies Targeting the E Protein

Neutralizing antibodies targeting the E protein represent one of the core research directions for post-exposure prophylaxis and treatment of severe ZIKV infections. Antibodies targeting the quaternary conformational epitope at the E protein dimer interface are ZIKV-specific and associated with the lowest documented risk of ADE. The representative monoclonal antibody ZIKV-117 and the fully human monoclonal antibody ZIKV-195 exhibit broad and potent neutralizing activity against multiple ZIKV lineages and significantly reduce tissue damage and improve survival in murine models. Notably, the Fc LALA mutant of ZIKV-117 completely eliminates ADE risk while preserving its biological activity [108]. ZIKV-195 blocks viral entry through a dual mechanism: cross-linking E protein dimers to stabilize the pre-fusion conformation and directly shielding the fusion loop [146].

Antibodies targeting DIII of the E protein represent an early direction in the development of anti-ZIKV neutralizing antibodies. The representative mAbs ZV-54 and ZV-67 both target the LR epitope on DIII, display broad neutralizing activity against ZIKV, confer complete protection against lethal ZIKV infection in mice, and carry an extremely low risk of ADE [147]. The fully human IgG1 cross-neutralizing mAb C10 targets the flavivirus-conserved pre-fusion EDE1 epitope, which is present exclusively in the native pre-fusion E dimer conformation. It demonstrates neutralizing activity against all major ZIKV strains in vitro; in vivo, a single prophylactic dose provides long-term suppression of systemic viremia in mice without inducing ADE [148].

Additionally, antibody 4G2 targets the highly conserved fusion-loop epitope at the tip of DII of flavivirus E proteins and broadly recognizes multiple flaviviruses. It is characterized by an extremely high risk of ADE and weak neutralizing activity, making it the most commonly used standard positive control antibody in flavivirus ADE research [100]. ZIKV vaccine and therapeutic antibody development has largely focused on IgG. However, recent studies isolated a pentameric monoclonal IgM antibody, DH1017. IgM, from Zika-infected pregnant women. This antibody exhibits extremely potent neutralizing activity against multiple Zika strains (FRNT_50_ ≈ 12 pM) and does not cross-react with dengue virus [149]. Structural studies indicate that it targets a quaternary epitope on domain II at the envelope E protein dimer interface, binds up to 10 antigenic sites simultaneously in a decavalent manner, and acts by recognizing and aggregating virus particles, which cannot be achieved by IgG [149]. Functionally, DH1017. IgM does not mediate ADE in either primary monocytes or monocytic cell lines; its corresponding IgG variant not only shows reduced neutralizing activity but also induces ADE. In a lethal mouse challenge model, DH1017. IgM was more effective than the IgG variant in controlling viremia [149]. However, its short half-life requires antibody engineering to prolong its duration of action, and its efficacy in nonhuman primates remains to be validated.

Current studies on anti-Zika virus therapeutics rely predominantly on in vitro neutralization assays, structural characterization, and murine protection experiments, with notable gaps in validations based on nonhuman primate models, gestational infection contexts, and clinical ADE risk evaluation. Most candidate therapeutics are tested merely in immortalized cell lines and/or immunodeficient mice. Considering the marked structural disparities between rodent and human placentas, as well as the fact that productive infection in adult wild-type mice depends on IFN signaling blockade or high-dose viral inoculation, the evaluation of therapeutic efficacy is prone to experimental bias. Viral epitope localization is a critical determinant of antibody neutralization potency, protective breadth, ADE risk, in vivo protective efficacy, and clinical translational prospects. With the iterative advancement of cryo-electron microscopy and auxiliary technologies, an increasing number of optimal antiviral epitopes have been precisely identified. In addition, rational engineering of antibodies can enhance therapeutic efficacy, safety, and blood–brain barrier permeability without impairing antiviral potency. Such multi-technology-integrated strategies will remain a central research direction for the development of anti-Zika virus therapeutics.

### 4.2. Drugs Related to Non-Structural Proteins

Current research and development in this field primarily focuses on the NS2B-NS3 protease complex and NS5 protein, which have well-defined enzymatic activities, while proteins such as NS1, NS4A, and NS4B also show promising therapeutic potential [150].

#### 4.2.1. Inhibitors Targeting the NS2B-NS3 Protease Complex

Inhibitors targeting the NS2B-NS3 protease complex exert their antiviral effects primarily by inhibiting its enzymatic activity and blocking viral polyprotein processing. Researchers conducted multi-target molecular-docking-based virtual screening of 2263 plant secondary metabolites and identified 43 drug-like phytochemicals with excellent binding affinities to key ZIKV non-structural proteins. Notably, several highly active lead compounds were derived from common medicinal plants, including Glycyrrhiza glabra (licorice), Rosmarinus officinalis (rosemary), Actaea racemosa (black cohosh), and Echinacea purpurea (purple coneflower) [151].

Further studies demonstrated that liansinine exerts potent cell-type-independent anti-ZIKV activity in U251, BHK21, and Vero cell lines (EC_50_ = 0.73 μM). It is the only compound in its derivative series that can effectively penetrate the BBB; in a neonatal mouse model of lethal ZIKV infection, it conferred 100% survival and significantly inhibited viral replication in brain tissues [152].

In addition to natural products, synthetic small molecules also exhibit inhibitory activity against the ZIKV NS2B-NS3 protease. Compound **1**, identified through high-throughput screening of 467,000 compounds, showed IC_50_ values of 0.69–1.7 μM against Asian lineage ZIKV and 5.0 μM against the African lineage MR766 strain in Huh7 cells, with low cytotoxicity and a wide safety window [153].

Computational simulation studies revealed that the sulfonamide-containing naphthoquinone derivative PAV-05, multiple benzopyran derivatives, and the aryl benzoylhydrazide (ABH) derivative 11q all exhibit very high binding affinities to the ZIKV NS2B-NS3 protease [154,155,156,157]. Among them, 11q and the novel thiazolidinedione derivative GQ-396 are rare examples of a dual-target design in ZIKV antiviral drug development, simultaneously targeting both the NS2B-NS3 protease and the RNA-dependent RNA polymerase (RdRp) domain of NS5. Compared with single-target inhibitors, this dual-target synergistic strategy blocks two critical steps of the viral life cycle, thereby significantly reducing the risk of drug-resistant viral mutations [154,155].

Based on a drug repurposing strategy, researchers conducted systematic computational screening and validation of 20 approved HIV/HCV protease inhibitors and found that the FDA-approved anti-HCV drug paritaprevir exhibits strong and stable binding affinities to the NS2B-NS3 proteases of ZIKV, DENV, and WNV [158]. Similarly, the commonly used clinical antibiotic doxycycline almost completely eliminates ZIKV infection-induced cytopathic effect at a concentration of 40 μM [159]. The omega-3 fatty acid eicosapentaenoic acid (EPA) also possesses anti-ZIKV activity (IC_50_ = 0.42 μM) with a wide safety window, and exhibits broad-spectrum inhibitory activity against multiple enveloped viruses [160].

Translation of liensinine and related compounds faces substantial barriers: nonhuman primate (NHP) efficacy and pharmacokinetic (PK) data are lacking; safety during pregnancy and long-term safety have not been validated; and efficacious human exposure, dosing regimens, and reproductive toxicology remain unknown. These gaps make it difficult to directly extrapolate their efficacy in cell-based assays and neonatal mice to clinical settings. Most other NS2B-NS3 inhibitors are still supported primarily by computational or in vitro data. 11q and GQ-396 dually target NS2B-NS3 and NS5 RdRp, theoretically blocking two key steps and reducing the risk of resistance; however, current evidence is largely derived from computational simulations, and data on in vivo protection, exposure–response, safety window, CNS distribution, and pregnancy toxicology are lacking. Their off-target toxicity and druggability have not yet been evaluated. Repurposed drugs such as paritaprevir, doxycycline, and EPA have established clinical safety and pharmacokinetic profiles and may shorten development timelines, but it remains to be determined whether clinically achievable exposures can attain effective anti-ZIKV concentrations. This line of investigation should prioritize candidates that simultaneously exhibit in vivo protection, BBB penetration, a wide safety window, and a complete chain of evidence, while clearly distinguishing preclinical activity from clinical feasibility.

#### 4.2.2. Inhibitors Targeting the NS5 Protein

Inhibitors targeting the NS5 protein also hold considerable promise for development, with those targeting the RdRp domain representing the core of research in this field. These inhibitors exert their antiviral effects primarily by competitively binding to the RdRp active pocket and blocking viral RNA chain elongation. Various agents, including natural products, nucleoside analogs, and small-molecule compounds, have demonstrated considerable inhibitory potential [161,162,163,164].

The approved drug lapatinib exhibits potent anti-flavivirus activity, with an EC_50_ as low as 2 μM against ZIKV. Lapatinib possesses a dual antiviral mechanism: it inhibits the human epidermal growth factor receptor 2 downstream signaling pathway to regulate host fatty acid synthase function [165]. Ivermectin exhibits broad-spectrum anti-flavivirus activity by blocking the interaction between NS5 and importin α/β1, with potent efficacy against DENV-1 to DENV-4 and ZIKV [166]. Computational simulations revealed that the approved antifungal drug posaconazole potently inhibits viral RdRp enzymatic activity and significantly suppresses ZIKV replication in Huh-7 cells [162]. The synthetic deoxynucleoside triphosphates dBTP and dZTP bind potently to the NS5 RdRp domain, with binding free energies 22–26 kcal/mol more favorable than those of the natural substrate guanosine triphosphate, making them highly promising novel nucleoside lead compounds for anti-ZIKV drug development [167].

Through natural product library screening, researchers discovered that the tea-derived natural polyphenol theaflavin potently inhibits ZIKV NS5 methyltransferase (MTase) activity (IC_50_ = 10.10 μM), with an EC_50_ of 8.19 μM against ZIKV replication in Huh-7 cells and extremely low cytotoxicity (CC_50_ > 128 μM) [161]. SMU-1k, obtained through structural modification of the lead compound ZINC000060514583, specifically blocks the interaction between NS5 and signal transducer and activator of transcription 2 (STAT2), inhibits NS5-mediated proteasomal degradation of STAT2, and restores the antiviral activity of the host IFN-I signaling pathway. This class of compounds exerts antiviral effects by restoring the host innate immune response, with a low risk of drug resistance [168].

Inhibition of both functional domains of NS5 represents an important future research direction. Additionally, dual-targeting strategies against NS2B–NS3 and NS5 can reduce the risk of drug resistance, constituting another highly promising approach [154,155]. Repurposed drugs have well-characterized clinical safety and pharmacokinetic profiles. Compared with inhibitors with novel chemical structures, they can substantially reduce safety risks and shorten the development cycles of antiviral agents during clinical translation [158,159].

Evidence for NS5 inhibitors is likewise derived predominantly from cell-based or computational models, with insufficient data on in vivo protection, resistance barriers, and combination regimens. BBB penetration is limited, and CNS distribution assessments are lacking; data on pregnancy safety, reproductive toxicology, and NHP are also insufficient. Host-directed strategies such as SMU-1k require in vivo validation of immune safety, whereas dual-target strategies require establishment of in vivo exposure–response relationships. Agents that directly inhibit viral enzymes should be evaluated for resistance and broad-spectrum activity, while host-directed agents should be assessed for immune safety and durability; both require hierarchical evidence evaluation. Even favorable cellular IC50 values are insufficient to bridge to clinically deployable therapies.

#### 4.2.3. Inhibitors Targeting Other Non-Structural Proteins

In addition to NS2B-NS3 and NS5, other non-structural proteins of ZIKV also exhibit promising therapeutic potential, among which NS1 is an important yet underexploited target for antiviral drug development [169]. The NS1-targeting mAb 1G5.3 blocks NS1-mediated disruption of cellular permeability, exhibits broad-spectrum anti-flavivirus activity in vitro, and reduces viremia and improves survival in mice. Importantly, it avoids ADE because NS1 is not exposed on the viral surface [170]. In DENV-related studies, compounds targeting NS4A and NS4B have demonstrated antiviral activity: SBI0090799 inhibits viral replication by interacting with NS4A, whereas JNJ-1A suppresses NS4B activity [171,172]. Due to the high structural conservation among flaviviruses, some effective drugs developed for DENV and Japanese encephalitis virus (JEV) infections can be repurposed for anti-ZIKV research [173]. The pan-flavivirus protease inhibitor NSC135618, an allosteric inhibitor of the DENV-2 protease, exhibits inhibitory activity against ZIKV (EC_50_ = 1.0 μM), YFV (EC_50_ = 0.28 μM), and WNV (EC_50_ = 1.27 μM). It also blocks ZIKV replication in placental epithelial cells, suggesting potential for preventing vertical transmission [28].

NS1 targeting may circumvent ADE but remains underexploited, with limited in vivo data. NS4A and NS4B inhibitors are largely derived from DENV studies, and their ZIKV specificity and in vivo efficacy remain to be validated. NSC135618 shows potential for preventing vertical transmission but requires validation in pregnancy models, pharmacokinetic studies, and reproductive toxicology. Non-structural protein targets can be extended to non-surface proteins to reduce ADE risk; cross-flavivirus repurposing requires ZIKV-specific validation and evaluation in pregnancy settings.

### 4.3. mRNA and sfRNA as Potential Therapeutic Strategies

mRNA technology offers a transformative and versatile therapeutic platform for the prevention and control of emerging and re-emerging infectious diseases. Apart from the aforementioned mRNA vaccines, mRNA-encoded neutralizing antibodies represent another rapidly developing therapeutic approach [174]. In a mouse model of lethal ZIKV challenge, intramuscular administration of ZIKV-117 mRNA conferred robust protective efficacy, whether administered as pre-exposure prophylaxis or post-exposure treatment [175]. This platform overcomes the limitations of conventional monoclonal antibody (mAb) therapies, which rely on labor-intensive and time-consuming cell culture-based production, and provides a more rapid, scalable, and cell-free manufacturing route—a core advantage for the rapid response to emerging infectious disease outbreaks [174].

In addition to virus-targeted neutralizing antibody strategies, mRNA technology has been extended to host-directed broad-spectrum antiviral research. Building on the broad-spectrum antiviral phenotype observed in ISG15-deficient individuals, researchers constructed modISG10, an LNP-formulated mRNA combination encoding 10 interferon-stimulated genes. In vitro assays demonstrated that modISG10 significantly inhibits ZIKV infection through multigene synergy, thus overcoming the limitations of previous ZIKV-focused mRNA development, which was predominantly centered on vaccines and neutralizing antibodies [176].

Subgenomic flavivirus RNA (sfRNA) is a non-coding RNA generated by the incomplete degradation of viral genomic RNA by the host 5′→3′ exoribonuclease XRN1 during ZIKV infection. sfRNA facilitates ZIKV immune evasion by binding to and stabilizing the viral NS5 protein, thereby reducing STAT1 phosphorylation and inhibiting its nuclear translocation [92]. Furthermore, sfRNA acts as a key mediator of viral pathogenesis and mosquito-borne transmission, making it a highly promising target for anti-ZIKV drug development [177]. The biogenesis of ZIKV sfRNA depends entirely on the higher-order structure of xrRNA within the 3′ untranslated region (3′UTR). Disruption of the pseudoknot or critical folding motifs of this structure completely abolishes the nuclease resistance of xrRNA and drastically reduces sfRNA production during viral infection [178]. Another study demonstrated that abrogation of xrRNA1-dependent sfRNA1 synthesis in ZIKV results in tissue-specific attenuation of viral replication [179]. This represents an important direction for anti-ZIKV drug development and also provides a novel strategy for the rational design of live-attenuated ZIKV vaccines. Nevertheless, the in vivo safety and protective efficacy of such interventions remain key issues that must be prioritized for clinical translation. Antisense oligonucleotides (ASOs) are short oligonucleotides that bind to target RNAs through complementary base pairing and can inhibit viral gene expression via multiple mechanisms, such as RNase H-mediated target RNA degradation [180]. However, their application in targeting ZIKV sfRNA has not yet been reported. Therapeutic strategies targeting sfRNA encounter multiple technical hurdles. First, sfRNA arises from incomplete XRN1 degradation of highly structured xrRNA elements within the 3′ untranslated region (3′UTR). Its pseudoknot and multiloop structures confer robust nuclease resistance and form cryptic complex target sites, impeding efficient recognition and binding by conventional antisense oligonucleotides (ASOs) [181,182]. Second, substantial sequence heterogeneity exists in the flavivirus 3′ UTR across strains; ASOs tailored to one ZIKV lineage frequently fail against other lineages [183]. Third, ASO targeted delivery represents a major bottleneck. Tissues relevant to Zika pathogenesis, including the placenta and fetal central nervous system, are protected by multiple physiological barriers (cell membrane, blood–brain barrier, placental barrier), and no validated delivery platform can accomplish targeted delivery during infection [184]. Finally, sfRNA is a noncoding regulatory RNA that stabilizes NS5 and modulates host signaling pathways. sfRNA knockdown yields pleiotropic antiviral effects, complicating discrimination between antiviral activity and direct cytotoxicity [92]. Future sfRNA-targeted drug development requires resolving the fine structural accessibility of xrRNA in infected cells, conjugating therapeutics to targeted nanoparticles or exosomes, and performing subsequent preclinical validation [185].

## 5. Conclusions

Accumulating studies have confirmed that the structural and non-structural proteins of ZIKV serve as key molecules driving the development of CZS and GBS. This review mainly illustrates the molecular mechanisms whereby viral proteins synergistically mediate host barrier disruption, neurodevelopmental damage, and antagonism of IFN-I immunity. We also provide a critical appraisal of ADE, a major bottleneck, as well as corresponding emerging vaccine counter-strategies. In the field of antiviral research, this paper dissects the translational prospects of candidate therapeutics targeting the E protein, NS2B-NS3 protease, and NS5 RdRp. Furthermore, we discuss the application potential of liansinine, which is capable of crossing the blood–brain barrier, together with dual-target inhibitory strategies.

Despite these advances, three major scientific challenges remain to be addressed in this research field. At the pathogenic mechanism level, most existing investigations focus on the functions of individual viral proteins. The synergistic regulatory networks formed by viral proteins and the full landscape of virus–host interactions have not been fully delineated. Receptor redundancy participates in ZIKV barrier crossing and tissue tropism modulation, and the physiological roles of receptors such as AXL remain controversial. For vaccine development, ADE continues to represent a primary limiting factor for ZIKV vaccine design. Although the molecular basis of ADE has been characterized via in vitro and animal experiments, pre-existing DENV immunity in human populations exerts bidirectional effects including ADE and cross-protection, and the mechanisms governing such duality are incompletely understood. Strategies including epitope screening, epitope modification, and heterologous antigen presentation can mitigate ADE risks in preclinical studies; nevertheless, the real-world clinical risks across diverse vaccine platforms require further validation. Due to ethical and practical constraints, pregnant women are difficult to recruit into clinical trials. Alternative evaluation systems based on CoP combined with nonhuman primate bridging studies still need further optimization. From the perspective of antiviral translation, small-molecule inhibitors, neutralizing antibodies, and drug-repurposing candidates targeting E protein, NS2B-NS3 and NS5 exhibit antiviral activities in vitro and in rodent models. Liansinine and dual-target inhibitors offer new insights to overcome viral drug resistance. However, the vast majority of candidate molecules lack systematic assessments in nonhuman primate models concerning pharmacokinetics, blood–brain barrier penetration, placental transfer, as well as reproductive and teratogenic toxicity.

Looking ahead, cutting-edge technologies such as CRISPR-mediated genome editing, single-cell and spatial transcriptomics, and cryo-electron microscopy will facilitate systematic dissection of the interaction network between ZIKV proteins and host factors. Artificial intelligence-aided drug discovery is expected to expedite the development of candidate antivirals and prophylactic vaccines. Meanwhile, continuous refinement of lateral-flow immunoassays and LAMP-based diagnostic platforms will provide robust tools for global prevention and control of ZIKV.

## Data Availability

No new data were created or analyzed in this study. Data sharing is not applicable to this article.

## Abbreviations

The following abbreviations are used in this manuscript:

3′UTR	3′ untranslated region
ABH	Aryl benzoylhydrazide
ADE	Antibody-dependent enhancement
ALPHAscreen	Amplified luminescent proximity homogeneous assay
ASOs	Antisense oligonucleotides
BBB	Blood–brain barrier
cGAS	Cyclic GMP-AMP synthase
CZS	Congenital Zika syndrome
D-box	Destruction box
DENV	Dengue virus
DHCR7	7-dehydrocholesterol reductase
DIII	Domain III
EDEs	Envelope dimeric epitopes
EGCG	Epigallocatechin gallate
EPA	Eicosapentaenoic acid
Ery-Est	Erythromycin estolate
FL	Fusion loop
FLEs	Fusion loop epitopes
G79	Glycine 79
GAS6	Growth arrest-specific gene 6
GBS	Guillain–Barré syndrome
IC_50_	Half-maximal inhibitory concentrations
IC_90_	90% inhibitory concentrations
IL-10	Interleukin-10
IRF3	Interferon regulatory factor 3
JEV	Japanese encephalitis virus
LNP	Lipid nanoparticle
mAb	Monoclonal antibody
miRNAs	MicroRNAs
MTase	Methyltransferase
NETs	Neutrophil extracellular traps
NF-κB	Nuclear factor kappa-B
NMD	Nonsense-mediated mRNA decay
NPCs	Neural progenitor cells
NS1	Non-structural protein 1
NS2A	Non-structural protein 2A
NS2B	Non-structural protein 2B
NS3	Non-structural protein 3
NS4A	Non-structural protein 4A
NS4B	Non-structural protein 4B
NS5	Non-structural protein 5
PAF1C	Polymerase II-associated factor 1 complex
PARP1	Poly(ADP-ribose) polymerase 1
PEX19	Peroxisomal biogenesis factor 19
prM	Pre-membrane protein
PRRs	Pattern recognition receptors
PS	Phosphatidylserine
R&D	Research and development
R78	Arginine 78
RdRp	RNA-dependent RNA polymerase
ROS	Reactive oxygen species
SERCA	Sarco/endoplasmic reticulum Ca^2+^-ATPase
sfRNA	Subgenomic flavivirus RNA
STAT1	Signal transducer and activator of transcription 1
STAT2	Signal transducer and activator of transcription 2
STING	Stimulator of interferon genes
TBK1	TANK-binding kinase 1
TNTs	Tunneling nanotubes
TRIM25	Tripartite motif-containing protein 25
TRIM7	Tripartite motif-containing protein 7
VE	Vascular endothelial
VLP	Virus-like particle
WNV	West Nile virus
ZIKV	Zika virus

## Appendix A

**Vaccine** **Platform**	**Representative Vaccine**	**Antigen**	**Safety Profile**	**Phase**	**Advantages**	**Limitations**	**Ref.**
**Inactivated** **Vaccine**	ZPIV	Whole virus	No vaccine-related adverse events of special interest were reported in any subjects	Phase I	Mature manufacturing technology, favorable safety profile, and excellent storage stability	Adjuvant-dependent immunogenicity; requires multiple doses; high production cost	[186]
**Live** **Attenuated Vaccine**	rZIKV /D4Δ30-713	rZIKV /D4Δ30–713	Safe and well-tolerated in healthy adults	Phase I	Induces durable immune response without adjuvants or multiple doses	Risk of insufficient immune stimulation due to over-attenuation; potential safety risks; contraindicated in immunocompromised populations and pregnant individuals	[187]
**mRNA** **Vaccine**	mRNA-1893	prM-E	Most vaccine-related adverse events were grade 1 or 2, with an overall favorable tolerability profile	Phase II	Short R&D cycle; no risk of host genome integration; favorable safety profile	Poor thermal stability; strict cold chain requirement; need for booster immunization	[188]
**DNA Vaccine**	VRC5283	prM-E	Favorable safety profile with no serious adverse events reported; incidence of local reactions <10%	Phase II	High chemical stability; low production cost	Weaker immunogenicity compared to mRNA and inactivated vaccines; susceptibility to degradation by endogenous nucleases in vivo	[189]
**Viral Vector Vaccine**	MV-ZIKA-RSP	prM/E	Favorable safety profile; pre-existing anti-vector immunity may reduce vaccine immunogenicity	Phase I	Induces comprehensive humoral and cellular immune responses; controllable production cost	Contraindicated in immunocompromised populations and pregnant individuals	[190]
**Virus-Like Particle (VLP) Vaccine**	EDIII -QβVLP	EDIII	No risk of viral infection and replication; negligible ADE risk	Preclinical	Viral genome-free; excellent safety profile; accurate display of conformational epitopes	High production cost; difficulty in large-scale manufacturing; slow clinical translation progress	[129]
**Recombinant Subunit** **Vaccine**	E80	E	Low-level ADE in vitro; no infection enhancement in vivo	Preclinical	Superior safety profile; controllable production cost	Low intrinsic immunogenicity; requires potent adjuvants; need for multiple doses	[191]

## References

[1] de Jong H.K., Grobusch M.P. (2025). Zika virus: An overview update. Curr. Opin. HIV AIDS.

[2] Oliveira F.B.C., Freire V., Coelho S.V.A., Meuren L.M., Palmeira J.D.F., Cardoso A.L., Neves F.A.R., Ribeiro B.M., Argañaraz G.A., Arruda L.B. (2023). ZIKV Strains Elicit Different Inflammatory and Anti-Viral Responses in Microglia Cells. Viruses.

[3] Cugola F.R., Fernandes I.R., Russo F.B., Freitas B.C., Dias J.L., Guimarães K.P., Benazzato C., Almeida N., Pignatari G.C., Romero S. (2016). The Brazilian Zika virus strain causes birth defects in experimental models. Nature.

[4] Dick G.W., Kitchen S.F., Haddow A.J. (1952). Zika virus. I. Isolations and serological specificity. Trans. R. Soc. Trop. Med. Hyg..

[5] Fauci A.S., Morens D.M. (2016). Zika Virus in the Americas--Yet Another Arbovirus Threat. N. Engl. J. Med..

[6] Ikejezie J., Shapiro C.N., Kim J., Chiu M., Almiron M., Ugarte C., Espinal M.A., Aldighieri S. (2017). Zika Virus Transmission—Region of the Americas, May 15, 2015–December 15, 2016. MMWR Morb. Mortal. Wkly. Rep..

[7] Guo Z., Jing W., Liu J., Liu M. (2022). The global trends and regional differences in incidence of Zika virus infection and implications for Zika virus infection prevention. PLoS Negl. Trop. Dis..

[8] Baker R.E., Mahmud A.S., Miller I.F., Rajeev M., Rasambainarivo F., Rice B.L., Takahashi S., Tatem A.J., Wagner C.E., Wang L.F. (2022). Infectious disease in an era of global change. Nat. Rev. Microbiol..

[9] Sirohi D., Chen Z., Sun L., Klose T., Pierson T.C., Rossmann M.G., Kuhn R.J. (2016). The 3.8 Å resolution cryo-EM structure of Zika virus. Science.

[10] Sirohi D., Kuhn R.J. (2017). Zika Virus Structure, Maturation, and Receptors. J. Infect. Dis..

[11] Shan C., Xia H., Haller S.L., Azar S.R., Liu Y., Liu J., Muruato A.E., Chen R., Rossi S.L., Wakamiya M. (2020). A Zika virus envelope mutation preceding the 2015 epidemic enhances virulence and fitness for transmission. Proc. Natl. Acad. Sci. USA.

[12] He M.J., Wang H.J., Yan X.L., Lou Y.N., Song G.Y., Li R.T., Zhu Z., Zhang R.R., Qin C.F., Li X.F. (2023). Key Residue in the Precursor Region of M Protein Contributes to the Neurovirulence and Neuroinvasiveness of the African Lineage of Zika Virus. J. Virol..

[13] Shang J., Zhou C., He M., Huang X.Y., Qin C.F., Wu A. (2025). Mutation S139N on Zika virus prM protein shifts immune response from Asian to contemporary strain. Brain Behav. Immun..

[14] Avila-Perez G., Nogales A., Park J.-G., Marquez-Jurado S., Iborra F.J., Almazan F., Martinez-Sobrido L. (2019). A natural polymorphism in Zika virus NS2A protein responsible of virulence in mice. Sci. Rep..

[15] Márquez-Jurado S., Nogales A., Ávila-Pérez G., Iborra F.J., Martínez-Sobrido L., Almazán F. (2018). An Alanine-to-Valine Substitution in the Residue 175 of Zika Virus NS2A Protein Affects Viral RNA Synthesis and Attenuates the Virus In Vivo. Viruses.

[16] Peng N.Y.G., Amarilla A.A., Hugo L.E., Modhiran N., Sng J.D.J., Slonchak A., Watterson D., Setoh Y.X., Khromykh A.A. (2022). The distinguishing NS5-M114V mutation in American Zika virus isolates has negligible impacts on virus replication and transmission potential. PLoS Negl. Trop. Dis..

[17] Annamalai A.S., Pattnaik A., Sahoo B.R., Muthukrishnan E., Natarajan S.K., Steffen D., Vu H.L.X., Delhon G., Osorio F.A., Petro T.M. (2017). Zika Virus Encoding Nonglycosylated Envelope Protein Is Attenuated and Defective in Neuroinvasion. J. Virol..

[18] Guo M., Hui L., Nie Y., Tefsen B., Wu Y. (2021). ZIKV viral proteins and their roles in virus-host interactions. Sci. China Life Sci..

[19] Shi H., Feng S., Naeem A., Huang B., Tan W., Zheng L., Liang X.J., Yang M., Huang Y. (2025). Bioprofiling and vaccine development for Zika virus. Sci. China Life Sci..

[20] Baric T.J., Reneer Z.B. (2024). Animal Models, Therapeutics, and Vaccine Approaches to Emerging and Re-Emerging Flaviviruses. Viruses.

[21] Yin W., Zhou R., Zhang W., Li Y. (2026). Zika virus entry inhibitors. Bioorganic Med. Chem..

[22] Miner J.J., Diamond M.S. (2017). Zika Virus Pathogenesis and Tissue Tropism. Cell Host Microbe.

[23] Wells M.F., Salick M.R., Wiskow O., Ho D.J., Worringer K.A., Ihry R.J., Kommineni S., Bilican B., Klim J.R., Hill E.J. (2016). Genetic Ablation of AXL Does Not Protect Human Neural Progenitor Cells and Cerebral Organoids from Zika Virus Infection. Cell Stem Cell.

[24] Dejnirattisai W., Supasa P., Wongwiwat W., Rouvinski A., Barba-Spaeth G., Duangchinda T., Sakuntabhai A., Cao-Lormeau V.M., Malasit P., Rey F.A. (2016). Dengue virus sero-cross-reactivity drives antibody-dependent enhancement of infection with zika virus. Nat. Immunol..

[25] Gordon A., Gresh L., Ojeda S., Katzelnick L.C., Sanchez N., Mercado J.C., Chowell G., Lopez B., Elizondo D., Coloma J. (2019). Prior dengue virus infection and risk of Zika: A pediatric cohort in Nicaragua. PLoS Med..

[26] Rodriguez-Barraquer I., Costa F., Nascimento E.J.M., Nery N.J., Castanha P.M.S., Sacramento G.A., Cruz J., Carvalho M., De Olivera D., Hagan J.E. (2019). Impact of preexisting dengue immunity on Zika virus emergence in a dengue endemic region. Science.

[27] Katzelnick L.C., Narvaez C., Arguello S., Lopez Mercado B., Collado D., Ampie O., Elizondo D., Miranda T., Bustos Carillo F., Mercado J.C. (2020). Zika virus infection enhances future risk of severe dengue disease. Science.

[28] Bernatchez J.A., Tran L.T., Li J., Luan Y., Siqueira-Neto J.L., Li R. (2020). Drugs for the Treatment of Zika Virus Infection. J. Med. Chem..

[29] Gardinali N.R., Marchevsky R.S., Vieira Y.C., Pelajo-Machado M., Kugelmeier T., Melgaço J.G., Castro M.P., de Oliveira J.M., Pinto M.A. (2025). Congenital Zika virus infection in laboratory animals: A comparative review highlights translational studies on the maternal-foetal interface. Mem. Inst. Oswaldo Cruz.

[30] WHO (2018). Zika Virus Research and Development (R&D) Roadmap.

[31] Hazin A.N., Poretti A., Di Cavalcanti Souza Cruz D., Tenorio M., van der Linden A., Pena L.J., Brito C., Gil L.H., de Barros Miranda-Filho D., Marques E.T. (2016). Computed Tomographic Findings in Microcephaly Associated with Zika Virus. N. Engl. J. Med..

[32] Cao-Lormeau V.M., Blake A., Mons S., Lastère S., Roche C., Vanhomwegen J., Dub T., Baudouin L., Teissier A., Larre P. (2016). Guillain-Barré Syndrome outbreak associated with Zika virus infection in French Polynesia: A case-control study. Lancet.

[33] da Silva I.R.F., Frontera J.A., Bispo de Filippis A.M., Nascimento O. (2017). Neurologic Complications Associated With the Zika Virus in Brazilian Adults. JAMA Neurol..

[34] Pindi C., Chirasani V.R., Senapati S. (2021). Identifying crucial E-protein residues responsible for unusual stability of Zika virus envelope. Biophys. J..

[35] Ma X., Yuan Z., Yi Z. (2022). Identification and characterization of key residues in Zika virus envelope protein for virus assembly and entry. Emerg. Microbes Infect..

[36] Kaur G., Pant P., Bhagat R., Seth P. (2023). Zika virus E protein modulates functions of human brain microvascular endothelial cells and astrocytes: Implications on blood-brain barrier properties. Front. Cell. Neurosci..

[37] Lo N.T.N., Roodsari S.Z., Tin N.L., Wong M.P., Biering S.B., Harris E. (2022). Molecular Determinants of Tissue Specificity of Flavivirus Nonstructural Protein 1 Interaction with Endothelial Cells. J. Virol..

[38] Bhardwaj U., Singh S.K. (2023). Zika virus NS1 suppresses VE-cadherin via hsa-miR-29b-3p/DNMT3b/MMP-9 pathway in human brain microvascular endothelial cells. Cell. Signal..

[39] Bhardwaj U., Singh S.K. (2021). Zika Virus NS1 Suppresses VE-Cadherin and Claudin-5 via hsa-miR-101-3p in Human Brain Microvascular Endothelial Cells. Mol. Neurobiol..

[40] Puerta-Guardo H., Biering S.B., de Sousa F.T.G., Shu J., Glasner D.R., Li J., Blanc S.F., Beatty P.R., Harris E. (2022). Flavivirus NS1 Triggers Tissue-Specific Disassembly of Intercellular Junctions Leading to Barrier Dysfunction and Vascular Leak in a GSK-3β-Dependent Manner. Pathogens.

[41] Michita R.T., Tran L.B., Bark S.J., Kumar D., Toner S.A., Jose J., Mysorekar I.U., Narayanan A. (2025). Zika Virus NS1 Drives Tunneling Nanotube Formation for Mitochondrial Transfer and Stealth Transmission in Trophoblasts. Nat. Commun..

[42] Nowakowski T.J., Pollen A.A., Di Lullo E., Sandoval-Espinosa C., Bershteyn M., Kriegstein A.R. (2016). Expression Analysis Highlights AXL as a Candidate Zika Virus Entry Receptor in Neural Stem Cells. Cell Stem Cell.

[43] Perera-Lecoin M., Meertens L., Carnec X., Amara A. (2013). Flavivirus Entry Receptors: An Update. Viruses.

[44] Giraldo M.I., Xia H., Aguilera-Aguirre L., Hage A., van Tol S., Shan C., Xie X., Sturdevant G.L., Robertson S.J., McNally K.L. (2020). Envelope protein ubiquitination drives entry and pathogenesis of Zika virus. Nature.

[45] Carbaugh D.L., Baric R.S., Lazear H.M. (2019). Envelope Protein Glycosylation Mediates Zika Virus Pathogenesis. J. Virol..

[46] Zhang L., Shim B.S., Kitzmiller C., Kwon Y.C., Richard A.S., Farzan M.R., Choe H. (2025). Variation in virion phosphatidylserine content drives differential GAS6 binding among closely related flaviviruses. J. Virol..

[47] Richard A.S., Shim B.S., Kwon Y.C., Zhang R., Otsuka Y., Schmitt K., Berri F., Diamond M.S., Choe H. (2017). AXL-dependent infection of human fetal endothelial cells distinguishes Zika virus from other pathogenic flaviviruses. Proc. Natl. Acad. Sci. USA.

[48] Jemielity S., Wang J.J., Chan Y.K., Ahmed A.A., Li W., Monahan S., Bu X., Farzan M., Freeman G.J., Umetsu D.T. (2013). TIM-family proteins promote infection of multiple enveloped viruses through virion-associated phosphatidylserine. PLoS Pathog..

[49] Strange D.P., Jiyarom B., Pourhabibi Zarandi N., Xie X., Baker C., Sadri-Ardekani H., Shi P.Y., Verma S. (2019). Axl Promotes Zika Virus Entry and Modulates the Antiviral State of Human Sertoli Cells. mBio.

[50] Li F., Wang P.R., Qu L.B., Yi C.H., Zhang F.C., Tang X.P., Zhang L.G., Chen L. (2017). AXL is not essential for Zika virus infection in the mouse brain. Emerg. Microbes Infect..

[51] Hastings A.K., Yockey L.J., Jagger B.W., Hwang J., Uraki R., Gaitsch H.F., Parnell L.A., Cao B., Mysorekar I.U., Rothlin C.V. (2017). TAM Receptors Are Not Required for Zika Virus Infection in Mice. Cell Rep..

[52] Yu J., Zheng Y.M., Sheridan M.A., Li P., Ezashi T., Roberts R.M., Liu S.L. (2025). Autophagy-mediated downregulation of AXL and TIM-1 promotes sustained Zika virus infection. Proc. Natl. Acad. Sci. USA.

[53] Santos C.N.O., Magalhães L.S., Fonseca A.B.L., Bispo A.J.B., Porto R.L.S., Alves J.C., Dos Santos C.A., de Carvalho J.V., da Silva A.M., Teixeira M.M. (2023). Association between genetic variants in TREM1, CXCL10, IL4, CXCL8 and TLR7 genes with the occurrence of congenital Zika syndrome and severe microcephaly. Sci. Rep..

[54] Azamor T., Cunha D.P., da Silva A.M.V., Bezerra O.C.L., Ribeiro-Alves M., Calvo T.L., Kehdy F.S.G., Manta F.S.N., Pinto T.G.T., Ferreira L.P. (2021). Congenital Zika Syndrome Is Associated With Interferon Alfa Receptor 1. Front. Immunol..

[55] Gomes J.A., Sgarioni E., Boquett J.A., Terças-Trettel A.C.P., da Silva J.H., Ribeiro B.F.R., Galera M.F., de Oliveira T.M., Carvalho de Andrade M.D.F., Carvalho I.F. (2021). Association between Genetic Variants in NOS2 and TNF Genes with Congenital Zika Syndrome and Severe Microcephaly. Viruses.

[56] Fontaine K.A., Leon K.E., Khalid M.M., Tomar S., Jimenez-Morales D., Dunla M., Kaye J.A., Shah P.S., Finkbeiner S., Krogan N.J. (2018). The Cellular NMD Pathway Restricts Zika Virus Infection and Is Targeted by the Viral Capsid Protein. mBio.

[57] Zeng J., Dong S., Luo Z., Xie X., Fu B., Li P., Liu C., Yang X., Chen Y., Wang X. (2020). The Zika Virus Capsid Disrupts Corticogenesis by Suppressing Dicer Activity and miRNA Biogenesis. Cell Stem Cell.

[58] He J., Yang L.P., Chang P.X., Yang S.X., Wang Y., Lin S.L., Tang Q.Y., Zhang Y.J. (2023). Zika Virus Induces Degradation of the Numb Protein Required through Embryonic Neurogenesis. Viruses.

[59] Wang Y., Ma Z.H., Hassaan N.A., Hao G., Wang P.H., Yao M., Wu C., Chen M., Xing L. (2025). Zika virus capsid protein impairs neural differentiation. Int. J. Biol. Macromol..

[60] Martins A.S., Carvalho F.A., Nascimento A.R., Silva N.M., Rebelo T.V., Faustino A.F., Enguita F.J., Huber R.G., Santos N.C., Martins I.C. (2024). Zika virus capsid protein closed structure modulates binding to host lipid systems. Protein Sci..

[61] Li H., Saucedo-Cuevas L., Yuan L., Ross D., Johansen A., Sands D., Stanley V., Guemez-Gamboa A., Gregor A., Evans T. (2019). Zika Virus Protease Cleavage of Host Protein Septin-2 Mediates Mitotic Defects in Neural Progenitors. Neuron.

[62] Apridita Sebastian W., Shiraishi H., Shimizu N., Umeda R., Lai S., Ikeuchi M., Morisaki I., Yano S., Yoshimura A., Hanada R. (2022). Ankle2 deficiency-associated microcephaly and spermatogenesis defects in zebrafish are alleviated by heterozygous deletion of vrk1. Biochem. Biophys. Res. Commun..

[63] Link N., Chung H., Jolly A., Withers M., Tepe B., Arenkiel B.R., Shah P.S., Krogan N.J., Aydin H., Geckinli B.B. (2019). Mutations in ANKLE2, a ZIKA Virus Target, Disrupt an Asymmetric Cell Division Pathway in Drosophila Neuroblasts to Cause Microcephaly. Dev. Cell.

[64] Liang Q., Luo Z., Zeng J., Chen W., Foo S.S., Lee S.A., Ge J., Wang S., Goldman S.A., Zlokovic B.V. (2016). Zika Virus NS4A and NS4B Proteins Deregulate Akt-mTOR Signaling in Human Fetal Neural Stem Cells to Inhibit Neurogenesis and Induce Autophagy. Cell Stem Cell.

[65] Chen W., Li Y., Yu X., Wang Z., Wang W., Rao M., Li Y., Luo Z., Zhang Q., Liu J. (2023). Zika virus non-structural protein 4B interacts with DHCR7 to facilitate viral infection. Virol. Sin..

[66] Anton A., Mazeaud C., Freppel W., Gilbert C., Tremblay N., Sow A.A., Roy M., Rodrigue-Gervais I.G., Chatel-Chaix L. (2021). Valosin-containing protein ATPase activity regulates the morphogenesis of Zika virus replication organelles and virus-induced cell death. Cell. Microbiol..

[67] Li P., Wu J., Liu S., Lu R., Jiang H., Wang N., Luo M., Guo L., Xiao J., Bu L. (2022). The RNA polymerase of cytoplasmically replicating Zika virus binds with chromatin DNA in nuclei and regulates host gene transcription. Proc. Natl. Acad. Sci. USA.

[68] Lebeau G., Hoareau M., Rivière S., El Safadi D., Da Silva C.R., Krejbich-Trotot P., Viranaicken W. (2024). Cell cycle and mitosis progression during ZIKA virus infection: The viral non-structural protein NS5 as a master regulator of the APC/cyclosome?. Biochimie.

[69] Pérez-Yanes S., Lorenzo-Sánchez I., Cabrera-Rodríguez R., García-Luis J., Trujillo-González R., Estévez-Herrera J., Valenzuela-Fernández A. (2024). The ZIKV NS5 Protein Aberrantly Alters the Tubulin Cytoskeleton, Induces the Accumulation of Autophagic p62 and Affects IFN Production: HDAC6 Has Emerged as an Anti-NS5/ZIKV Factor. Cells.

[70] Steiner J.P., Bachani M., Malik N., Li W., Tyagi R., Sampson K., Abrams R.P.M., Kousa Y., Solis J., Johnson T.P. (2023). Neurotoxic properties of the Zika virus envelope protein. Exp. Neurol..

[71] Guo Y.Q., Bao L.L., Xu Y.F., Li F.D., Lv Q., Fan F.Y., Qin C.A. (2021). The Ablation of Envelope Protein Glycosylation Enhances the Neurovirulence of ZIKV and Cell Apoptosis in Newborn Mice. J. Immunol. Res..

[72] Lebeau G., El Safadi D., Hoarau M., Meilhac O., Krejbich-Trotot P., Viranaicken W. (2024). Zika virus restriction of host antioxidant response is mediated by intracellular NS1 and reveals its ability to upregulate Bach1 expression. Biochem. Biophys. Res. Commun..

[73] de Siqueira Santos R., Rochael N.C., Mattos T.R.F., Silva M.F.F.e., Linhares-Lacerda L., de Oliveira L.T., Cunha M.S., Mohana-Borges R., Gomes T.A., Barbosa-Silva M.C. (2023). Peripheral nervous system is injured by neutrophil extracellular traps (NETs) elicited by nonstructural (NS) protein-1 from Zika virus. FASEB J..

[74] Huang S., Shi P.-D., Fan X.-X., Yang Y., Qin C.-F., Zhao H., Shi L., Ci Y. (2024). The glycosylation deficiency of flavivirus NS1 attenuates virus replication through interfering with the formation of viral replication compartments. J. Biomed. Sci..

[75] Huang C., Jiang T., Pan W., Feng T., Zhou X., Wu Q., Ma F., Dai J. (2024). Ubiquitination of NS1 Confers Differential Adaptation of Zika Virus in Mammalian Hosts and Mosquito Vectors. Adv. Sci..

[76] Wang S., Tang S., Zhou Y., An Q., Du M., Yang X., Zou P., Tang L., Yu Y. (2025). Zika virus non-structural protein NS2A mediated endoplasmic reticulum stress through interacting with Sarco/endoplasmic reticulum Ca^2+^-ATPase 2. J. Virol..

[77] Yang W., Li H., Wang S., Huang R., Zhang Y., Guo M., Huang L., Li S., Yang R., Zhao D. (2025). Zika virus disrupts steroidogenesis and impairs spermatogenesis by stalling the translation of CYP17A1 mRNA. Nat. Commun..

[78] Han X., Wang J., Yang Y., Qu S., Wan F., Zhang Z., Wang R., Li G., Cong H. (2021). Zika virus infection induced apoptosis by modulating the recruitment and activation of pro-apoptotic protein Bax. J. Virol..

[79] Coldbeck-Shackley R.C., Eyre N.S., Beard M.R. (2020). The Molecular Interactions of ZIKV and DENV with the Type-I IFN Response. Vaccines.

[80] Creisher P.S., Klein S.L. (2024). Pathogenesis of viral infections during pregnancy. Clin. Microbiol. Rev..

[81] Airo A.M., Felix-Lopez A., Mancinelli V., Evseev D., Lopez-Orozco J., Shire K., Paszkowski P., Frappier L., Magor K.E., Hobman T.C. (2022). Flavivirus Capsid Proteins Inhibit the Interferon Response. Viruses.

[82] Farelo M.A., Korrou-Karava D., Brooks K.F., Russell T.A., Maringer K., Mayerhofer P.U. (2022). Dengue and Zika Virus Capsid Proteins Contain a Common PEX19-Binding Motif. Viruses.

[83] Sui L.Y., Zhao Y.H., Wang W.F., Chi H.M., Tian T., Wu P., Zhang J.L., Zhao Y.C., Wei Z.K., Hou Z.J. (2023). Flavivirus prM interacts with MDA5 and MAVS to inhibit RLR antiviral signaling. Cell Biosci..

[84] Yoon K.-J., Song G., Qian X., Pan J., Xu D., Rho H.-S., Kim N.-S., Habela C., Zheng L., Jacob F. (2017). Zika-Virus-Encoded NS2A Disrupts Mammalian Cortical Neurogenesis by Degrading Adherens Junction Proteins. Cell Stem Cell.

[85] Xia H., Luo H., Shan C., Muruato A.E., Nunes B.T.D., Medeiros D.B.A., Zou J., Xie X., Giraldo M.I., Vasconcelos P.F.C. (2018). An evolutionary NS1 mutation enhances Zika virus evasion of host interferon induction. Nat. Commun..

[86] Ding Q., Gaska J.M., Douam F., Wei L., Kim D., Balev M., Heller B., Ploss A. (2018). Species-specific disruption of STING-dependent antiviral cellular defenses by the Zika virus NS2B3 protease. Proc. Natl. Acad. Sci. USA.

[87] Lee J.Y., Nguyen T.T.N., Myoung J. (2020). Zika Virus-Encoded NS2A and NS4A Strongly Downregulate NF-κB Promoter Activity. J. Microbiol. Biotechnol..

[88] Lee J.K., Shin O.S. (2023). Zika virus modulates mitochondrial dynamics, mitophagy, and mitochondria-derived vesicles to facilitate viral replication in trophoblast cells. Front. Immunol..

[89] Zeng Q., Liu J., Hao C., Zhang B., Zhang H. (2023). Making sense of flavivirus non-strctural protein 1 in innate immune evasion and inducing tissue-specific damage. Virus Res..

[90] Sarratea M.B., Alberti A.S., Redolfi D.M., Truant S.N., Iannantuono Lopez L.V., Bivona A.E., Mariuzza R.A., Fernández M.M., Malchiodi E.L. (2023). Zika virus NS4B protein targets TANK-binding kinase 1 and inhibits type I interferon production. Biochim. Biophys. Acta Gen. Subj..

[91] Li Y., Li Z., Zou H., Zhou P., Huo Y., Fan Y., Liu X., Wu J., Li G., Wang X. (2024). A conserved methyltransferase active site residue of Zika virus NS5 is required for the restriction of STING activation and interferon expression. J. Gen. Virol..

[92] Slonchak A., Wang X., Aguado J., Sng J.D.J., Chaggar H., Freney M.E., Yan K., Torres F.J., Amarilla A.A., Balea R. (2022). Zika virus noncoding RNA cooperates with the viral protein NS5 to inhibit STAT1 phosphorylation and facilitate viral pathogenesis. Sci. Adv..

[93] Espada C.E., da Rocha E.L., Ricciardi-Jorge T., Dos Santos A.A., Soares Z.G., Malaquias G., Patrício D.O., Gonzalez Kozlova E., Dos Santos P.F., Bordignon J. (2024). ISG15/USP18/STAT2 is a molecular hub regulating IFN I-mediated control of Dengue and Zika virus replication. Front. Immunol..

[94] Parisien J.P., Lenoir J.J., Alvarado G., Horvath C.M. (2022). The Human STAT2 Coiled-Coil Domain Contains a Degron for Zika Virus Interferon Evasion. J. Virol..

[95] Fanunza E., Carletti F., Quartu M., Grandi N., Ermellino L., Milia J., Corona A., Capobianchi M.R., Ippolito G., Tramontano E. (2021). Zika virus NS2A inhibits interferon signaling by degradation of STAT1 and STAT2. Virulence.

[96] Wu Y., Liu Q., Zhou J., Xie W., Chen C., Wang Z., Yang H., Cui J. (2017). Zika virus evades interferon-mediated antiviral response through the co-operation of multiple nonstructural proteins in vitro. Cell Discov..

[97] Sawant J., Patil A., Kurle S. (2023). A Review: Understanding Molecular Mechanisms of Antibody-Dependent Enhancement in Viral Infections. Vaccines.

[98] Iqbal N.T., Sawatzki K., Ahmed K., Tisoncik-Go J., Smith E., Voss K., Cornelius J., Wang L., Spaulding A.B., Serebryannyy L. (2025). Metagenomic investigation of febrile illness in Pakistan reveals global transmission and co-circulation of Zika and Dengue viruses. medRxiv.

[99] Mukhopadhyay S., Kuhn R.J., Rossmann M.G. (2005). A structural perspective of the flavivirus life cycle. Nat. Rev. Microbiol..

[100] Yan Y., Wang J., Yang H., Yang Y., Yuan L., Tang C., Zhou Y., Huang Q., Yu W., Liang X. (2025). The modified mRNA vaccine protects immunocompromised AG129 mice from lethal challenge and multi-tissue infection by Zika virus. Emerg. Microbes Infect..

[101] Filatov A.V., Byazrova M.G., Sukhova M.M., Prilipov A.G. (2026). Molecular Mechanisms of Antibody-Dependent Enhancement of Viral Infection. Biochem. Biokhimiia.

[102] Guzman M.G., Halstead S.B., Artsob H., Buchy P., Farrar J., Gubler D.J., Hunsperger E., Kroeger A., Margolis H.S., Martínez E. (2010). Dengue: A continuing global threat. Nat. Rev. Microbiol..

[103] Shukla R., Ramasamy V., Shanmugam R.K., Ahuja R., Khanna N. (2020). Antibody-Dependent Enhancement: A Challenge for Developing a Safe Dengue Vaccine. Front. Cell. Infect. Microbiol..

[104] Narayan R., Tripathi S. (2020). Intrinsic ADE: The Dark Side of Antibody Dependent Enhancement During Dengue Infection. Front. Cell. Infect. Microbiol..

[105] Katzelnick L.C., Gresh L., Halloran M.E., Mercado J.C., Kuan G., Gordon A., Balmaseda A., Harris E. (2017). Antibody-dependent enhancement of severe dengue disease in humans. Science.

[106] Cabral-Miranda G., Lim S.M., Mohsen M.O., Pobelov I.V., Roesti E.S., Heath M.D., Skinner M.A., Kramer M.F., Martina B.E.E., Bachmann M.F. (2019). Zika Virus-Derived E-DIII Protein Displayed on Immunologically Optimized VLPs Induces Neutralizing Antibodies without Causing Enhancement of Dengue Virus Infection. Vaccines.

[107] Stettler K., Beltramello M., Espinosa D.A., Graham V., Cassotta A., Bianchi S., Vanzetta F., Minola A., Jaconi S., Mele F. (2016). Specificity, cross-reactivity, and function of antibodies elicited by Zika virus infection. Science.

[108] Sapparapu G., Fernandez E., Kose N., Bin C., Fox J.M., Bombardi R.G., Zhao H., Nelson C.A., Bryan A.L., Barnes T. (2016). Neutralizing human antibodies prevent Zika virus replication and fetal disease in mice. Nature.

[109] Yang M., Dent M., Lai H., Sun H., Chen Q. (2017). Immunization of Zika virus envelope protein domain III induces specific and neutralizing immune responses against Zika virus. Vaccine.

[110] Lin H.H., Yip B.S., Huang L.M., Wu S.C. (2018). Zika virus structural biology and progress in vaccine development. Biotechnol. Adv..

[111] Liu T., Li M., Tian Y., Dong Y., Liu N., Wang Z., Zhang H., Zheng A., Cui C. (2024). Immunogenicity and safety of a self-assembling ZIKV nanoparticle vaccine in mice. Int. J. Pharm..

[112] Qi J., He Y., Shen L., Yu W., Hu T. (2021). Conjugation of Hemoglobin and Mannan Markedly Improves the Immunogenicity of Domain III of the Zika Virus E Protein: Structural and Immunological Study. Bioconjug. Chem..

[113] Rong H., Qi M., Pan J., Sun Y., Gao J., Zhang X., Li W., Zhang B., Zhang X.E., Cui Z. (2022). Self-Assembling Nanovaccine Confers Complete Protection Against Zika Virus Without Causing Antibody-Dependent Enhancement. Front. Immunol..

[114] Pattnaik A., Sahoo B.R., Struble L.R., Borgstahl G.E.O., Zhou Y., Franco R., Barletta R.G., Osorio F.A., Petro T.M., Pattnaik A.K. (2023). A Ferritin Nanoparticle-Based Zika Virus Vaccine Candidate Induces Robust Humoral and Cellular Immune Responses and Protects Mice from Lethal Virus Challenge. Vaccines.

[115] Yen L.C., Chen H.W., Ho C.L., Lin C.C., Lin Y.L., Yang Q.W., Chiu K.C., Lien S.P., Lin R.J., Liao C.L. (2023). Neutralizing antibodies targeting a novel epitope on envelope protein exhibited broad protection against flavivirus without risk of disease enhancement. J. Biomed. Sci..

[116] Dai L., Xu K., Li J., Huang Q., Song J., Han Y., Zheng T., Gao P., Lu X., Yang H. (2021). Protective Zika vaccines engineered to eliminate enhancement of dengue infection via immunodominance switch. Nat. Immunol..

[117] Cuevas-Juárez E., Liñan-Torres A., Hernández C., Kopylov M., Potter C.S., Carragher B., Ramírez O.T., Palomares L.A. (2023). Mimotope discovery as a tool to design a vaccine against Zika and dengue viruses. Biotechnol. Bioeng..

[118] Rothen D.A., Dutta S.K., Krenger P.S., Pardini A., Vogt A.S., Josi R., Lieknina I., Osterhaus A., Mohsen M.O., Vogel M. (2024). Preclinical Development of a Novel Zika Virus-like Particle Vaccine in Combination with Tetravalent Dengue Virus-like Particle Vaccines. Vaccines.

[119] Georgiev G.I., Malonis R.J., Wirchnianski A.S., Wessel A.W., Jung H.S., Cahill S.M., Nyakatura E.K., Vergnolle O., Dowd K.A., Cowburn D. (2022). Resurfaced ZIKV EDIII nanoparticle immunogens elicit neutralizing and protective responses in vivo. Cell Chem. Biol..

[120] Lin H.H., Yang S.P., Tsai M.J., Lin G.C., Wu H.C., Wu S.C. (2019). Dengue and Zika Virus Domain III-Flagellin Fusion and Glycan-Masking E Antigen for Prime-Boost Immunization. Theranostics.

[121] Slon-Campos J.L., Dejnirattisai W., Jagger B.W., López-Camacho C., Wongwiwat W., Durnell L.A., Winkler E.S., Chen R.E., Reyes-Sandoval A., Rey F.A. (2019). A protective Zika virus E-dimer-based subunit vaccine engineered to abrogate antibody-dependent enhancement of dengue infection. Nat. Immunol..

[122] Zhao F., Xu Y., Liu N., Lv D., Chen Y., Liu Z., Jin X., Xiao M., Lavillette D., Zhong J. (2023). Extracellular vesicles from Zika virus-infected cells display viral E protein that binds ZIKV-neutralizing antibodies to prevent infection enhancement. EMBO J..

[123] Berneck B.S., Rockstroh A., Fertey J., Grunwald T., Ulbert S. (2020). A Recombinant Zika Virus Envelope Protein with Mutations in the Conserved Fusion Loop Leads to Reduced Antibody Cross-Reactivity upon Vaccination. Vaccines.

[124] Lai H., Paul A.M., Sun H., He J., Yang M., Bai F., Chen Q. (2018). A plant-produced vaccine protects mice against lethal West Nile virus infection without enhancing Zika or dengue virus infectivity. Vaccine.

[125] Fink A.L., Williams K.L., Harris E., Alvine T.D., Henderson T., Schiltz J., Nilles M.L., Bradley D.S. (2017). Dengue virus specific IgY provides protection following lethal dengue virus challenge and is neutralizing in the absence of inducing antibody dependent enhancement. PLoS Negl. Trop. Dis..

[126] O’Donnell K.L., Meberg B., Schiltz J., Nilles M.L., Bradley D.S. (2019). Zika Virus-Specific IgY Results Are Therapeutic Following a Lethal Zika Virus Challenge without Inducing Antibody-Dependent Enhancement. Viruses.

[127] Peng Z.-Y., Yang S., Lu H.-Z., Wang L.-M., Li N., Zhang H.-T., Xing S.-Y., Du Y.-N., Deng S.-Q. (2024). A review on Zika vaccine development. Pathog. Dis..

[128] Liu X., Li Z., Li X., Wu W., Jiang H., Zheng Y., Zhou J., Ye X., Lu J., Wang W. (2024). A single-dose circular RNA vaccine prevents Zika virus infection without enhancing dengue severity in mice. Nat. Commun..

[129] Côrtes N., Lira A., Silva J.D.Q., Carvalho E., Prates-Syed W.A., Hamaguchi B., Durães-Carvalho R., Balan A., Câmara N.O.S., Cabral-Marques O. (2025). A VLPs based vaccine protects against Zika virus infection and prevents cerebral and testicular damage. npj Vaccines.

[130] Wu B., Qi Z., Qian X. (2023). Recent Advancements in Mosquito-Borne Flavivirus Vaccine Development. Viruses.

[131] Ferguson N.M., Cucunubá Z.M., Dorigatti I., Nedjati-Gilani G.L., Donnelly C.A., Basáñez M.G., Nouvellet P., Lessler J. (2016). EPIDEMIOLOGY. Countering the Zika epidemic in Latin America. Science.

[132] Ethics Working Group on ZIKV Research and Pregnancy (2017). Ethics, pregnancy, and ZIKV vaccine research & development. Vaccine.

[133] Krubiner C.B., Faden R.R., Karron R.A., Little M.O., Lyerly A.D., Abramson J.S., Beigi R.H., Cravioto A.R., Durbin A.P., Gellin B.G. (2021). Pregnant women & vaccines against emerging epidemic threats: Ethics guidance for preparedness, research, and response. Vaccine.

[134] Tanriover M.D., Poulimeneas D., Tsopela G.C., Kopsidas I., Argyropoulos C., Di Marzo R., Valdenmaiier O., Jakobsen S.F., Valle Simon P., Carcas-Sansuan A.J. (2026). Barriers and Opportunities to Include Underrepresented Population Groups in Vaccine Trials: Cross-Sectional, Observational, Online Survey Study From the VACCELERATE Research Network. JMIR Public Health Surveill..

[135] Katzelnick L.C., Coloma J., Harris E. (2017). Dengue: Knowledge gaps, unmet needs, and research priorities. Lancet Infect. Dis..

[136] Plotkin S.A. (2010). Correlates of protection induced by vaccination. Clin. Vaccine Immunol. CVI.

[137] Abbink P., Larocca R.A., De La Barrera R.A., Bricault C.A., Moseley E.T., Boyd M., Kirilova M., Li Z., Ng’ang’a D., Nanayakkara O. (2016). Protective efficacy of multiple vaccine platforms against Zika virus challenge in rhesus monkeys. Science.

[138] Abbink P., Larocca R.A., Visitsunthorn K., Boyd M., De La Barrera R.A., Gromowski G.D., Kirilova M., Peterson R., Li Z., Nanayakkara O. (2017). Durability and correlates of vaccine protection against Zika virus in rhesus monkeys. Sci. Transl. Med..

[139] Roy A., Liu Q., Yang Y., Debnath A.K., Du L. (2024). Envelope Protein-Targeting Zika Virus Entry Inhibitors. Int. J. Mol. Sci..

[140] Carneiro B.M., Batista M.N., Braga A.C.S., Nogueira M.L., Rahal P. (2016). The green tea molecule EGCG inhibits Zika virus entry. Virology.

[141] Mounce B.C., Cesaro T., Carrau L., Vallet T., Vignuzzi M. (2017). Curcumin inhibits Zika and chikungunya virus infection by inhibiting cell binding. Antivir. Res..

[142] Pitts J., Hsia C.Y., Lian W., Wang J., Pfeil M.P., Kwiatkowski N., Li Z., Jang J., Gray N.S., Yang P.L. (2019). Identification of small molecule inhibitors targeting the Zika virus envelope protein. Antivir. Res..

[143] Elfiky A.A., Ibrahim I.M. (2021). Zika virus envelope—Heat shock protein A5 (GRP78) binding site prediction. J. Biomol. Struct. Dyn..

[144] Wang X., Xia S., Zou P., Lu L. (2019). Erythromycin Estolate Inhibits Zika Virus Infection by Blocking Viral Entry as a Viral Inactivator. Viruses.

[145] Bifani A.M., Chan K.W.K., Borrenberghs D., Tan M.J.A., Phoo W.W., Watanabe S., Goethals O., Vasudevan S.G., Choy M.M. (2023). Therapeutics for flaviviral infections. Antivir. Res..

[146] Long F., Doyle M., Fernandez E., Miller A.S., Klose T., Sevvana M., Bryan A., Davidson E., Doranz B.J., Kuhn R.J. (2019). Structural basis of a potent human monoclonal antibody against Zika virus targeting a quaternary epitope. Proc. Natl. Acad. Sci. USA.

[147] Zhao H., Fernandez E., Dowd K.A., Speer S.D., Platt D.J., Gorman M.J., Govero J., Nelson C.A., Pierson T.C., Diamond M.S. (2016). Structural Basis of Zika Virus-Specific Antibody Protection. Cell.

[148] Schramm N.J., Kovarova M., Manzoor S., Cockrell A.S., Spagnuolo R.A., Amblard F., Bassit L., Schinazi R.F., Baric R.S., Wahl A. (2025). Envelope-dimer epitope 1 (EDE1) antibody (C10) treatment significantly reduces Zika virus replication in the male and female reproductive tracts. J. Virol..

[149] Singh T., Hwang K.K., Miller A.S., Jones R.L., Lopez C.A., Dulson S.J., Giuberti C., Gladden M.A., Miller I., Webster H.S. (2022). A Zika virus-specific IgM elicited in pregnancy exhibits ultrapotent neutralization. Cell.

[150] Sadeer N.B., El Kalamouni C., Khalid A., Abdalla A.N., Zengin G., Khoa Bao L.V., Mahomoodally M.F. (2023). Secondary metabolites as potential drug candidates against Zika virus, an emerging looming human threat: Current landscape, molecular mechanism and challenges ahead. J. Infect. Public Health.

[151] Byler K.G., Ogungbe I.V., Setzer W.N. (2016). In-silico screening for anti-Zika virus phytochemicals. J. Mol. Graph. Model..

[152] Sun S., Sun T., Lei X., Li F., Wang J., Zhou R., Li Q., Yi D., Wang J., Jin H. (2025). Neferine inhibits Zika virus replication by targeting viral protease. Phytomed. Int. J. Phytother. Phytopharm..

[153] Shin H.J., Kim M.H., Lee J.Y., Hwang I., Yoon G.Y., Kim H.S., Kwon Y.C., Ahn D.G., Kim K.D., Kim B.T. (2021). Structure-Based Virtual Screening: Identification of a Novel NS2B-NS3 Protease Inhibitor with Potent Antiviral Activity against Zika and Dengue Viruses. Microorganisms.

[154] Ralph de Oliveira I.L., da Silva Moura J.A., Recordon-Pinson P., Lagadec F., da Rosa M.M., Gonçalves S.M.C., Viana D.C.F., de Moraes Gomes P.A.T., da Rocha Pitta M.G., de Melo Rêgo M.J.B. (2025). Novel Thiazolidinedione Derivatives as Potential ZIKV Antiviral Inhibitors. Microorganisms.

[155] Yadav R.P., Jena N.R. (2025). Aryl benzoyl hydrazide derivatives as potent inhibitors of the NS2B-NS3 protease and RNA-dependent RNA polymerase of the zika virus. Silico Pharmacol..

[156] Acharya S.S., Rout P.R., Barad L.M., Dansana J., Gadtya A., Meher B.R., Parida B.B. (2025). Visible light-driven photocatalyst-free cascade synthesis of functionalized benzopyrans as inhibitors of Chikungunya nsPs and Zika NS2B-NS3. Org. Biomol. Chem..

[157] Cirne-Santos C.C., Gonzaga D.T.G., de Resende G.O., de Castro Goncalves M., da Silva Andrade A., Furtado Pacheco P.A., Dos Santos-Rodrigues A., Rabelo V.W., Abreu P.A., Faria R.X. (2025). PAV-05 Naphthoquinone Potently Inhibit Zika Virus Replication in Infected Cells. Curr. Top. Med. Chem..

[158] Chong Teoh T., Al-Harbi S.J., Abdulrahman A.Y., Rothan H.A. (2021). Doxycycline Interferes with Zika Virus Serine Protease and Inhibits Virus Replication in Human Skin Fibroblasts. Molecules.

[159] Yadav R.P., Jena N.R. (2025). Paritaprevir as a pan-antiviral against different flaviviruses. Front. Mol. Biosci..

[160] Feng Y., Qiu S., Zou S., Li R., Chen H., Chen K., Ma J., Liu J., Lai X., Liu S. (2025). Antiviral activity of eicosapentaenoic acid against zika virus and other enveloped viruses. Front. Pharmacol..

[161] Song W., Zhang H., Zhang Y., Chen Y., Lin Y., Han Y., Jiang J. (2021). Identification and Characterization of Zika Virus NS5 Methyltransferase Inhibitors. Front. Cell. Infect. Microbiol..

[162] Chen Y., Chi X., Zhang H., Zhang Y., Qiao L., Ding J., Han Y., Lin Y., Jiang J. (2023). Identification of Potent Zika Virus NS5 RNA-Dependent RNA Polymerase Inhibitors Combining Virtual Screening and Biological Assays. Int. J. Mol. Sci..

[163] Yao G., Yu J., Lin C., Zhu Y., Duan A., Li M., Yuan J., Zhang J. (2022). Design, synthesis, and biological evaluation of novel 2’-methyl-2’-fluoro-6-methyl-7-alkynyl-7-deazapurine nucleoside analogs as anti-Zika virus agents. Eur. J. Med. Chem..

[164] Milisavljevic N., Konkolová E., Kozák J., Hodek J., Veselovská L., Sýkorová V., Čížek K., Pohl R., Eyer L., Svoboda P. (2021). Antiviral Activity of 7-Substituted 7-Deazapurine Ribonucleosides, Monophosphate Prodrugs, and Triphoshates against Emerging RNA Viruses. ACS Infect. Dis..

[165] Pant S., Jena N.R. (2025). Binding of Nucleotide Inhibitors to the NS5 RdRp of the ZIKA Virus in the Replication Initiation State. Curr. Med. Chem..

[166] Boldescu V., Behnam M.A.M., Vasilakis N., Klein C.D. (2017). Broad-spectrum agents for flaviviral infections: Dengue, Zika and beyond. Nat. Rev. Drug Discov..

[167] Pant S., Jena N.R. (2025). Computational predictions of artificial nucleoside triphosphates as potent inhibitors of RNA-dependent RNA polymerase of the ZIKA virus. Hum. Immunol..

[168] Lin R., Han J., He Y., Xie L., Gao T., Chen Y., Zhong Y., Ding Q., Cheng K., Yao X. (2025). Design, synthesis and biological evaluation of dapsone derivatives with broad-spectrum antiviral activity. Eur. J. Med. Chem..

[169] de Silva A.M., Rey F.A., Young P.R., Hilgenfeld R., Vasudevan S.G. (2018). Viral Entry and NS1 as Potential Antiviral Drug Targets. Adv. Exp. Med. Biol..

[170] Modhiran N., Song H., Liu L., Bletchly C., Brillault L., Amarilla A.A., Xu X., Qi J., Chai Y., Cheung S.T.M. (2021). A broadly protective antibody that targets the flavivirus NS1 protein. Science.

[171] Nobori H., Toba S., Yoshida R., Hall W.W., Orba Y., Sawa H., Sato A. (2018). Identification of Compound-B, a novel anti-dengue virus agent targeting the non-structural protein 4A. Antivir. Res..

[172] Hernandez-Morales I., Geluykens P., Clynhens M., Strijbos R., Goethals O., Megens S., Verheyen N., Last S., McGowan D., Coesemans E. (2017). Characterization of a dengue NS4B inhibitor originating from an HCV small molecule library. Antivir. Res..

[173] Stevens L.J., Pruijssers A.J., Lee H.W., Gordon C.J., Tchesnokov E.P., Gribble J., George A.S., Hughes T.M., Lu X., Li J. (2022). Mutations in the SARS-CoV-2 RNA-dependent RNA polymerase confer resistance to remdesivir by distinct mechanisms. Sci. Transl. Med..

[174] Singh D. (2026). mRNA-Encoded antibodies as a next-generation therapeutic paradigm: A rapid and adaptive platform for the prevention and treatment of emerging and re-emerging infectious diseases—A critical review. Immunol. Res..

[175] Erasmus J.H., Archer J., Fuerte-Stone J., Khandhar A.P., Voigt E., Granger B., Bombardi R.G., Govero J., Tan Q., Durnell L.A. (2020). Intramuscular Delivery of Replicon RNA Encoding ZIKV-117 Human Monoclonal Antibody Protects against Zika Virus Infection. Mol. Ther. Methods Clin. Dev..

[176] Akalu Y.T., Patel R.S., Taft J., Canas-Arranz R., Geltman R., Richardson A., Buta S., Martin-Fernandez M., Sazeides C., Pearl R.L. (2025). An mRNA-based broad-spectrum antiviral inspired by ISG15 deficiency protects against viral infections in vitro and in vivo. Sci. Transl. Med..

[177] Chen X., Li R.T., Chen R.Y., Shi P.D., Liu Z.X., Lou Y.N., Wu M., Zhang R.R., Tang W., Li X.F. (2024). The subgenomic flaviviral RNA suppresses RNA interference through competing with siRNAs for binding RISC components. J. Virol..

[178] Akiyama B.M., Laurence H.M., Massey A.R., Costantino D.A., Xie X., Yang Y., Shi P.Y., Nix J.C., Beckham J.D., Kieft J.S. (2016). Zika virus produces noncoding RNAs using a multi-pseudoknot structure that confounds a cellular exonuclease. Science.

[179] Sparks H., Monogue B., Akiyama B., Kieft J., Beckham J.D. (2020). Disruption of Zika Virus xrRNA1-Dependent sfRNA1 Production Results in Tissue-Specific Attenuated Viral Replication. Viruses.

[180] Crooke S.T., Baker B.F., Crooke R.M., Liang X.H. (2021). Antisense technology: An overview and prospectus. Nat. Rev. Drug Discov..

[181] Zhaguparov D., Zhao M., Sekar R.V., Woodside M.T. (2025). Identifying the interactions conferring functional mechanical rigidity on RNase-resistant RNA from Zika virus. Proc. Natl. Acad. Sci. USA.

[182] Niu X., Sun R., Chen Z., Yao Y., Zuo X., Chen C., Fang X. (2021). Pseudoknot length modulates the folding, conformational dynamics, and robustness of Xrn1 resistance of flaviviral xrRNAs. Nat. Commun..

[183] Slonchak A., Parry R., Pullinger B., Sng J.D.J., Wang X., Buck T.F., Torres F.J., Harrison J.J., Colmant A.M.G., Hobson-Peters J. (2022). Structural analysis of 3′UTRs in insect flaviviruses reveals novel determinants of sfRNA biogenesis and provides new insights into flavivirus evolution. Nat. Commun..

[184] Lewis E., Lee S.J., Park H.R. (2025). Placenta-derived extracellular vesicles in fetal health: Emerging insights into brain development and environmental interactions. Exp. Mol. Med..

[185] Doets K., Pijlman G.P. (2024). Subgenomic flavivirus RNA as key target for live-attenuated vaccine development. J. Virol..

[186] Koren M.A., Lin L., Eckels K.H., De La Barrera R., Dussupt V., Donofrio G., Sondergaard E.L., Mills K.T., Robb M.L., Lee C. (2023). Safety and immunogenicity of a purified inactivated Zika virus vaccine candidate in adults primed with a Japanese encephalitis virus or yellow fever virus vaccine in the USA: A phase 1, randomised, double-blind, placebo-controlled clinical trial. Lancet Infect. Dis..

[187] Zhong D., Kirkpatrick B., Pierce K., Patel U., Blunt M.R., Durbin A. (2022). 2138. Evaluating the Safety and Immunogenicity of the rZIKV/D4Δ30-713 Live Attenuated Chimeric Zika Candidate Vaccine in Healthy Flavivirus-Naïve Adults. Open Forum Infect. Dis..

[188] (2021). A Phase 2, Randomized, Observer-Blind, Placebo-Controlled, Dose Confirmation Study to Evaluate the Safety, Tolerability, and Immunogenicity of Zika Vaccine mRNA-1893 in Adults Aged 18 Through 65 Years and Living in Endemic and Non-Endemic Flavivirus Areas. https://trialscreen.org/trials/phase-2-zika-virus-vaccine-mrna-1893-adult-participants-living-endemic-trial-nct04917861.

[189] Gaudinski M.R., Houser K.V., Morabito K.M., Hu Z., Yamshchikov G., Rothwell R.S., Berkowitz N., Mendoza F., Saunders J.G., Novik L. (2018). Safety, tolerability, and immunogenicity of two Zika virus DNA vaccine candidates in healthy adults: Randomised, open-label, phase 1 clinical trials. Lancet.

[190] (2019). Observer Blinded, Randomized Trial to Evaluate Safety and Immunogenicity of a Novel Vaccine Formulation MV-ZIKA-RSP. https://trialscreen.org/trials/phase-1-safety-immunogenicity-novel-vaccine-formulation-mv-zika-rsp-v187-trial-nct04033068.

[191] Yang R., Liu Q., Pang W., Gao F., Liang H., Zhang W., Lin Y., Li M., Liu Z., Gao G.F. (2021). Two immunogenic recombinant protein vaccine candidates showed disparate protective efficacy against Zika virus infection in rhesus macaques. Vaccine.

